# Synthesis of a 1,2-Dithienylethene-Containing Donor-Acceptor Polymer via Palladium-Catalyzed Direct Arylation Polymerization (DArP)

**DOI:** 10.3390/molecules23040981

**Published:** 2018-04-23

**Authors:** Masayuki Wakioka, Natsumi Yamashita, Hiroki Mori, Yasushi Nishihara, Fumiyuki Ozawa

**Affiliations:** 1 International Research Center for Elements Science (IRCELS), Institute for Chemical Research, Kyoto University, Uji, Kyoto 611-0011, Japan; yamashita.natsumi.22v@st.kyoto-u.ac.jp; 2 Research Institute for Interdisciplinary Science, Okayama University, 3-1-1 Tsushimanaka, Kita-ku, Okayama 700-8530, Japan; h-mor@okayama-u.ac.jp (H.M.); ynishiha@okayama-u.ac.jp (Y.N.)

**Keywords:** direct arylation, polycondensation, palladium catalyst, conjugated polymer

## Abstract

This paper reports the synthesis of D-A polymers containing 1,2-dithienylethene (DTE) units via palladium-catalyzed direct arylation polymerization (DArP). The reaction of dibromoisoindigo (**1**-**Br**) and DTE (**2**-**H**), in the presence of Pd_2_(dba)_3_·CHCl_3_ (0.5 mol%), P(2-MeOC_6_H_4_)_3_ (**L1**) (2 mol%), pivalic acid (1 equiv) as catalyst precursors, and Cs_2_CO_3_ (3 equiv) as a base affords poly(**1**-*alt*-**2**) with a high molecular weight (*M*_n_ up to 44,900). Although, it has been known that monomers, with plural C–H bonds, tend to form insoluble materials via direct arylation at undesirable C–H positions; the reaction of **1**-**Br** and **2**-**H** cleanly proceeds without insolubilization. The resulting polymer has a well-controlled structure and exhibits good charge transfer characteristics in an organic field-effect transistor (OFET), compared to the polymer produced by Migita–Kosugi–Stille cross-coupling polymerization. The DArP product displays an ideal linear relationship in the current–voltage curve, whereas the Migita–Kosugi–Stille product shows a *V*_G_-dependent change in the charge mobility.

## 1. Introduction

π-Conjugated polymers with donor–acceptor combinations of repeated units (D-A polymers) often exhibit a high device performance in organic field-effect transistors (OFETs) and thin-film solar cells [1,2,3,4]. Thus far, such polymers have been prepared by Migita–Kosugi–Stille cross-coupling polymerization. However, this method needs toxic tin reagents for the monomer preparation, and the polymerization forms a highly toxic side product such as Me_3_SnBr [5]. It has also been implicated that the residual impurities that arise from tin reagents may deteriorate the device performance [6,7,8,9]. In this context, the palladium-catalyzed direct arylation polymerization (DArP), that proceeds via C–H bond activation, has recently emerged as a simple yet fundamental solution for these problems [10,11,12]. We have developed a highly efficient DArP catalyst using P(2-MeOC_6_H_4_)_3_ (**L1**) as a ligand to produce D-A polymers with a high molecular weight [13,14,15,16,17,18,19,20]. The resulting polymers have well-controlled structures without tin impurities, thereby leading to good material properties [16].

**Scheme 1:**
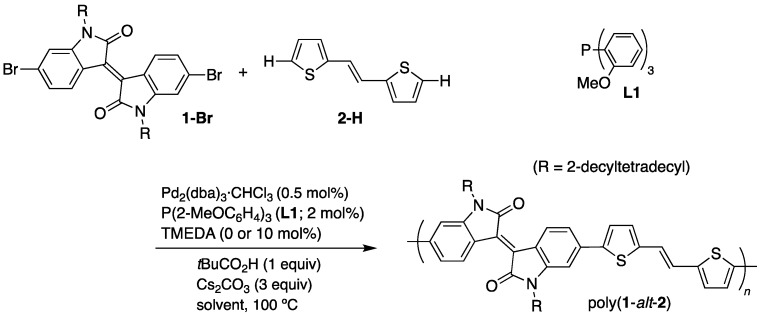
Synthesis of poly(**1**-*alt*-**2**) via DArP.

In this study, we examined the synthesis of a D-A polymer with 1,2-dithienylethene (DTE) as donor units; poly(**1**-*alt*-**2**) in Scheme 1. DTE-based D-A polymers, such as poly(**1**-*alt*-**2**), have been prepared by Migita–Kosugi–Stille cross-coupling polymerization and have proven to be competent compounds in OFETs [21,22,23]; however, their synthesis via DArP has been poorly successful [24]. This is probably due to the existence of plural C–H bonds in DTE. Direct arylation at undesirable C–H positions forms branching and cross-linking polymers, which eventually change to insoluble materials [25,26,27,28]. On the other hand, we found that the **L1**-based catalyst successfully produces the desired poly(**1**-*alt*-**2**) without formation of insoluble materials. The resulting polymer has a well-controlled structure and shows better charge transfer properties in OFET than Migita–Kosugi–Stille cross-coupling products.

## 2. Results and Discussion

### 2.1. Synthesis of Poly(**1**-alt-**2**)

First, we examined the DArP of dibromoisoindigo (**1**-**Br**) and DTE (**2**-**H**) in several types of solvents (Scheme 1). Table 1 summarizes the results. The reaction was conducted with Pd_2_(dba)_3_·CHCl_3_ (0.5 mol%), **L1** (2 mol%), pivalic acid (1 equiv) as catalyst precursors, and Cs_2_CO_3_ (3 equiv) as a base. When the reaction was carried out in toluene at 100 °C, the reaction mixture turned into a dark red mass in 5 h; however, Soxhlet extraction with *o*-Cl_2_C_6_H_4_ afforded poly(**1**-*alt*-**2**) (*M*_n_ = 17,000) in almost quantitative yield and no colored substance remained in the extraction thimble (run 1). THF was less effective as a solvent, giving a low-molecular weight polymer (*M*_n_ = 7400) (run 2). On the other hand, the molecular weight increased in 2-MeTHF (*M*_n_ = 22,700) (run 3), and when a low-molecular weight portion was removed by Soxhlet extraction with CHCl_3_, poly(**1**-*alt*-**2**) with *M*_n_ = 44,900 was obtained in a recovery rate of 63% (run 3a). Unlike the reaction in toluene, the reaction system retained liquidity in 2-MeTHF.

Next, we examined the synthesis of poly(**1**-*alt*-**2**) in the presence of *N*,*N*,*N′*,*N′*-tetramethylethylenediamine (TMEDA). We have documented that, while the reaction becomes slow, the combined use of **L1** and TMEDA effectively prevents the formation of structural defects, including homocoupling, branching, and cross-linking defects leading to insolubilization [18,19]. The reaction in toluene proceeded without the formation of a solid mass, and poly(**1**-*alt*-**2**) with *M*_n_ = 15,700 was produced in a high yield, in 24 h (run 4). On the other hand, the DArP in the presence of TMEDA was significantly slow in 2-MeTHF, and the molecular weight remained moderate, even after 96 h (run 5).

**Scheme 2:**
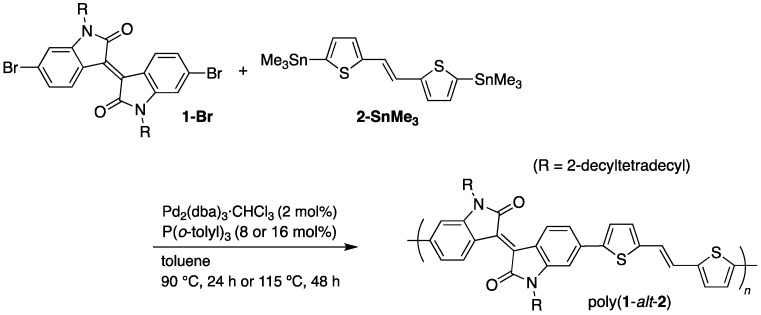
Synthesis of poly(**1**-*alt*-**2**) via Migita–Kosugi–Stille cross-coupling polymerization.

For comparison, poly(**1**-*alt*-**2**) was prepared by Migita–Kosugi–Stille cross-coupling polymerization (Scheme 2). Referring to the literature [22,23], the reactions of **1**-**Br** and **2**-**SnMe_3_** were examined in toluene under two catalytic conditions. The reaction in the presence of Pd_2_(dba)_3_·CHCl_3_ (2 mol%) and P(*o*-tolyl)_3_ (8 mol%) at 90 °C for 24 h yielded poly(**1**-*alt*-**2**) with moderate molecular weight (*M*_n_ = 17,700, PDI = 1.7) in quantitative yield [22]. Although the GPC molecular weight of the polymer prepared under those reactions conditions has been reported to be *M*_n_ = 98,800 (PDI = 3.1), this value has been estimated at 40 °C, using CHCl_3_ as the column eluate [22]. In contrast, we performed GPC calibration at 140 °C, using *o*-Cl_2_C_6_H_4_. Thus, we re-examined the polymer under the same GPC conditions using CHCl_3_ and confirmed that our product (*M*_n_ = 93,400, PDI = 4.6) has comparable molecular weight to that of the literature.

The synthesis of poly(**1**-*alt*-**2**) via Migita–Kosugi–Stille cross-coupling was also carried out under dilute conditions (monomers: 8 mM) using a large amount of P(*o*-tolyl)_3_ (16 mol%) at 115 °C for 48 h [23]. In this case, the resulting polymer was sparingly soluble and unable to be examined by GPC using *o*-Cl_2_C_6_H_4_ at 140 °C, whereas the molecular weight could be estimated at 150 °C using 1,2,4-Cl_3_C_6_H_3_ as the column eluate (*M*_n_ = 53,800, PDI = 2.8). The NMR analysis described below was performed for a soluble part of the product, prepared by Soxhlet extraction with CHCl_3_ (18% recovery, *M*_n_ = 24,400, PDI = 2.5).

### 2.2. ^1^H-NMR Analysis of Poly(**1**-alt-**2**)

**Figure 1:**
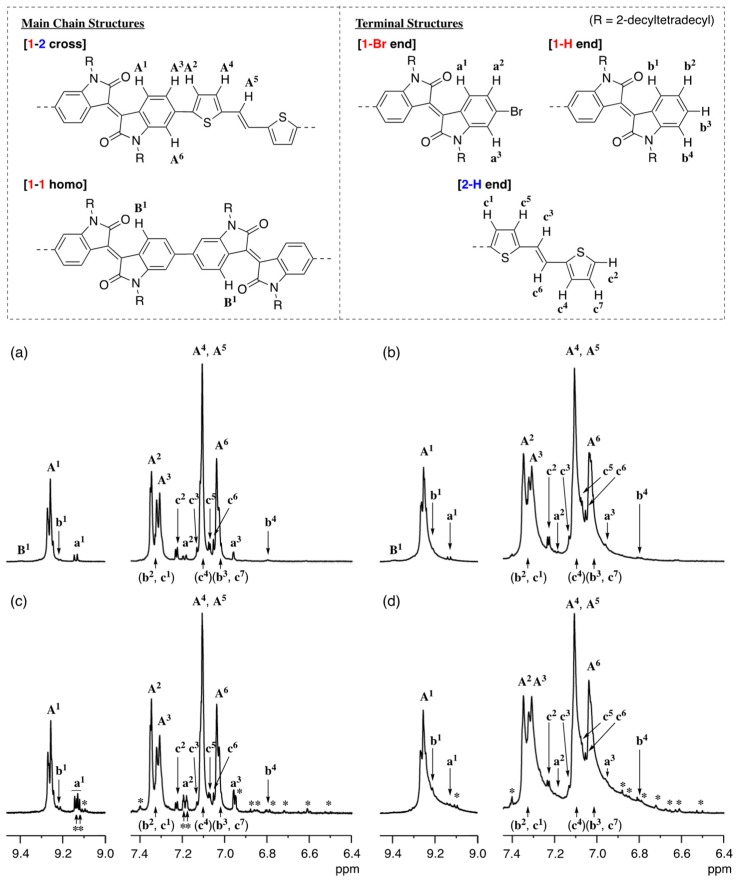
^1^H-NMR spectra of poly(**1**-*alt*-**2**) prepared via DArP (*M*_n_ = 15,700 (**a**) and *M*_n_ = 22,700 (**b**)) and Migita–Kosugi–Stille cross-coupling polymerization (*M*_n_ = 17,700 (**c**) and *M*_n_ = 24,400 (**d**)) in C_2_D_2_Cl_4_ at 130 °C (600 MHz). *: unidentified signals.

Figure 1 compares ^1^H-NMR spectra of the four types of poly(**1**-*alt*-**2**)s prepared by DArP (a and b) and Migita–Kosugi–Stille cross-coupling polymerization (c and d). The signal assignments are based on the spectra of model compounds of substructures and structural defects (see the Supplementary Materials). In Figure 1a—for the polymer of run 4, Table 1 (*M*_n_ = 15,700)—the spectrum consists of the main signals due to cross-coupling units (**A^1^**–**A^6^**) and small signals arise from three kinds of terminal groups (**a^1^**–**a^3^**, **b^1^**–**b^4^**, and **c^1^**–**c^7^**). Although the signal due to **1**-**1** homocoupling defects (**B**) is observed, its ratio is less than 0.1%. On the other hand, signals assignable to **2**-**2** homocoupling, branching, and cross-linking defects are not observed. Figure 1b, which corresponds to the polymer of run 3 in Table 1 (*M*_n_ = 22,700), displays the same signals as Figure 1a, although the relative intensity of each unit is changed, and the signals are broadened reflecting the increase in molecular weight. The number of **1**-**1** homocoupling defects (**B**) is estimated to be less than 0.1%, whereas other structural defects are not observed. Hence, the highly controlled structures of the DArP polymers have been confirmed.

On the other hand, Figure 1c,d for the Migita–Kosugi–Stille cross-coupling products (*M*_n_ = 17,700 and 24,400) contain many unidentified peaks in addition to the signals assigned to the core and terminal groups of poly(**1**-*alt*-**2**). Moreover, the signals are somewhat broadened as compared to the DArP products of comparable molecular weight.

### 2.3. Electronic Properties of Poly(**1**-alt-**2**)

**Figure 2:**
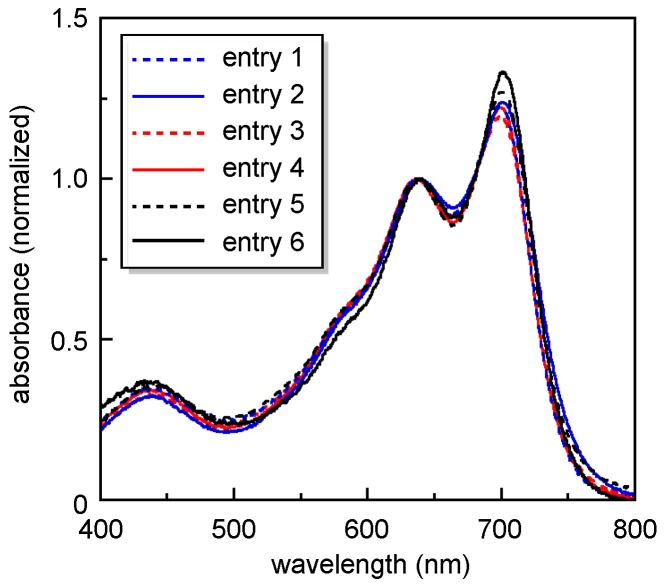
UV-vis spectra of poly(**1**-*alt*-**2**) in thin film. The entry number follows Table 2.

Table 2 summarizes the optical and electrochemical properties of poly(**1**-*alt*-**2**)s, in thin film, which were evaluated by UV–vis spectroscopy and cyclic voltammetry. The absorption spectra of poly(**1**-*alt*-**2**)s, listed in Table 2, are shown in Figure 2. All spectra consist of two absorption bands around 600–700 (band I) and 650–750 nm (band II). The band I arises from the intramolecular charge transfer (ICT) transitions in the donor–acceptor units, whereas the band II is induced for the aggregation of polymer chains through strong intermolecular interactions [29]. In Figure 2, the changes in the relative intensity, from band II to band I, are small. The maximum absorption wavelengths of polymers are approximately the same (λ_max_ = 638(3) and 700(2) nm). Moreover, the absorption onsets (λ_onset_) are within the range of 746–753 nm, showing that the optical band gaps are little affected by the polymerization methods, as well as the molecular weight (*E*_g_^opt^ = 1.66(1) eV). Indeed, the electronic properties evaluated by cyclic voltammetry are consistent for all polymers: *E*_HOMO_ = −5.34(2) eV, *E*_LUMO_ = −3.60(3) eV, *E*_g_^CV^ = 1.74(2) eV.

### 2.4. OFET Characteristics of Poly(**1**-alt-**2**)

OFET characteristics of the DArP polymers were compared with those of the Migita–Kosugi–Stille cross-coupling products. OFET devices were fabricated on an n^+^-Si/SiO_2_ substrate treated with *n*-octyltriethoxysilane (OTS) as a self-assembled monolayer (SAM). The active layer of poly(**1**-*alt*-**2**) was formed by spin-coating from a CHCl_3_ or *o*-Cl_2_C_6_H_4_ solution, and sequential thermal annealing was carried out at 240 °C for 30 min under an inert atmosphere. The solvents for spin-coating were changed according to the solubility of polymers. We confirmed that the solvents little affected the carrier mobility (Table S1).

Poly(**1**-*alt*-**2**) exhibited typical *p*-type characteristics under ambient conditions in the dark. Table 3 lists the hole mobility observed for the polymers shown in Table 2. The highest hole mobility was observed for the DArP polymer in entry 1 (*M*_n_ = 15,700, μ_h_ = 0.31 cm^2^ V^−1^ s^−1^), and this value was comparable to that for the Migita–Kosugi–Stille cross-coupling product with similar molecular weight in entry 5 (*M*_n_ = 17,700, μ_h_ = 0.28 cm^2^ V^−1^ s^−1^). On the other hand, the hole mobility for the other polymers was low (entries 2–4 and 6). It emerges that the hole mobility depends on the weight average molecular weight (*M*_w_), not on the number average molecular weight (*M*_n_). Indeed, the hole mobility decreases significantly with increasing the *M*_w_ values.

To understand the reasons behind the large variation in device performance depending on molecular weight, polymer thin films formed on an OTS-treated substrate were investigated by grazing incidence wide-angle X-ray scattering (GIWAXS) and atomic force microscopy (AFM). These investigations indicated that the hole mobility changes with the surface morphology rather than with the crystallinity (Figures S4–S6). The GIWAXS analysis revealed the predominant edge-on orientation on the substrate for all films. Moreover, in Table 3, the lamellar *d*-spacings (*d_l_*) and π–π stacking distances (*d*_π_) were very similar to each other, regardless of the *M*_w_ values and the polymerization methods *d_l_* = 21.4(7) Å and *d*_π_ = 3.75(4) Å. On the other hand, in the AFM images, the surface morphology clearly changed with *M*_w_, and the polymer with a lower *M*_w_ value tended to form a smoother surface. The polymers with a high *M*_w_ value in entries 4 and 6 showed several clear grain boundaries, suppressing the carrier transport efficiency.

**Figure 3:**
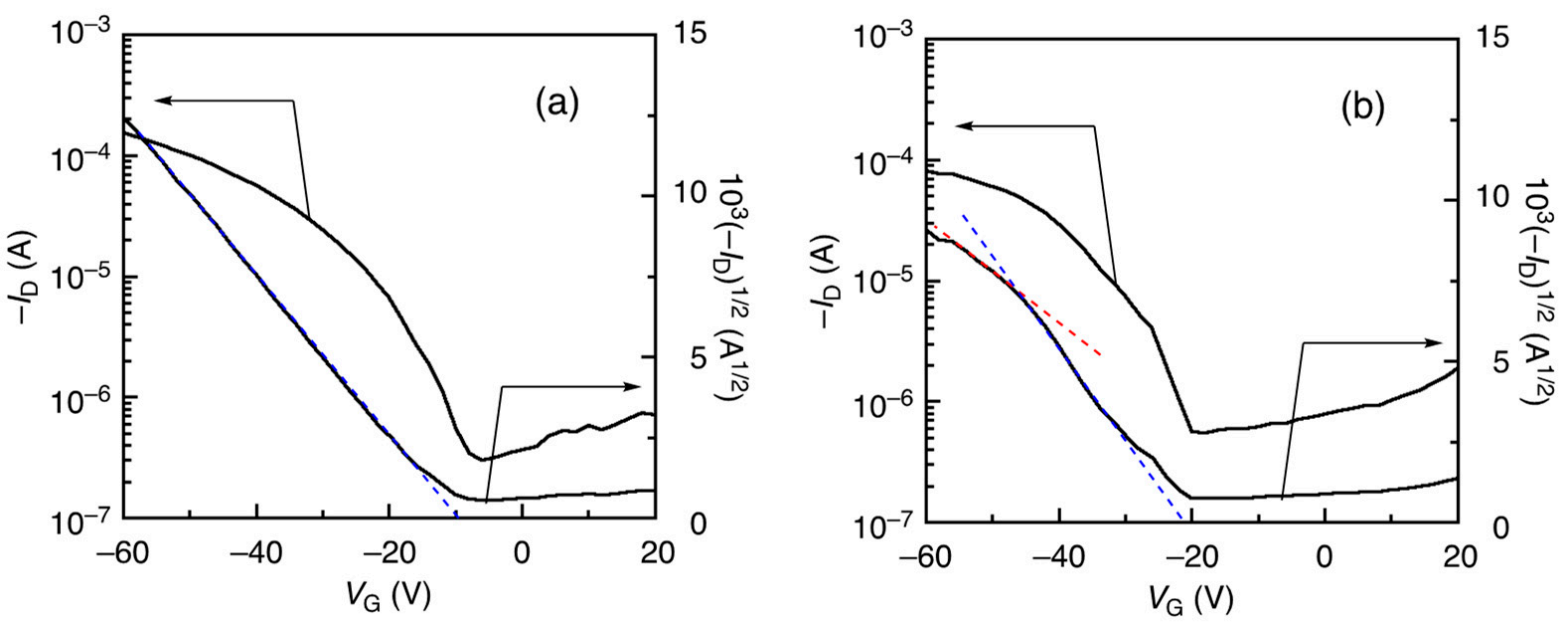
Charge transfer characteristics of the OFETs using poly(**1**-*alt*-**2**) of entries 1 (**a**) and 5 (**b**) in Table 3 (*V*_D_ = −60 V).

While the hole mobility was little affected by the polymerization methods, the charge transfer characteristics were clearly different for the DArP and Migita–Kosugi–Stille cross-coupling products. In Figure 3a, the DArP polymer (entry 1, Table 3) has the ideal transfer characteristics, showing a linear correlation in the current–voltage curve. Thus, this polymer exhibited a *V*_G_-independent change in the hole mobility. A similar trend was observed for all DArP products (see Figure S2). On the other hand, in Figure 3b, the Migita–Kosugi–Stille cross-coupling product (entry 5, Table 3) exhibited non-ideal double slope characteristics [30], which are often related to the contact resistance and/or the presence of carrier traps due to defects or impurities in the polymer transistors. The same phenomenon has already been documented for the poly(**1**-*alt*-**2**) prepared by Migita–Kosugi–Stille cross-coupling polymerization [22].

## 3. Conclusions

We have described the synthesis of D-A polymers containing 1,2-dithienylethene (DTE) units via palladium-catalyzed direct arylation polymerization (DArP). The reaction of dibromoisoindigo (**1**-**Br**) and DTE (**2**-**H**) using the catalytic system with P(2-MeOC_6_H_4_)_3_ (**L1**) as a ligand afforded the desired poly(**1**-*alt*-**2**) with a high molecular weight (*M*_n_ up to 44,900) and yield. The ^1^H-NMR analysis revealed a highly controlled structure for the resulting polymer. On the other hand, the ^1^H-NHR spectrum of the Migita–Kosugi–Stille cross-coupling polymerization product indicated the contamination of unknown structural defects. These polymers showed almost the same optical properties, independently of the polymerization methods. Moreover, they exhibited comparable charge mobility when the weight average molecular weight (*M*_w_) was similar. However, the charge transfer characteristics of the DArP product in OFET were superior to those of the Migita–Kosugi–Stille cross-coupling product. The DArP product displayed an ideal linear relationship in the current–voltage curve, whereas the Migita–Kosugi–Stille product showed a non-ideal double-slope curve. The so-called double-slope characteristics have been recognized to be a crucial problem in the practical applications of polymer-based OFETs [30]. Since the physical properties observed by spectroscopic and electrochemical methods were almost the same, the difference in charge transfer characteristics of these products is possibly due to the difference in structural defects. Moreover, there is a possibility that impurities arising from tin reagents cause the deterioration in device performance. Thus, the present study indicates that the highly selective synthesis of D-A polymers via DArP using P(2-MeOC_6_H_4_)_3_ (**L1**) as a ligand provides a simple but powerful approach for developing OFETs with good properties.

## 4. Materials and Methods

### 4.1. General Considerations

All manipulations were performed under a nitrogen atmosphere using Schlenk techniques or a glove box. Toluene, THF, and 2-MeTHF were dried over Na/Ph_2_CO, distilled, and stored over activated MS4A. Cs_2_CO_3_ was dried overnight at 120 °C under vacuum and handled in a glove box. PivOH was distilled and handled in a glove box. TMEDA was dried over CaH_2_ and distilled. **1**-**Br** [31], **2**-**H** [32], **2**-**SnMe_3_** [33], and Pd_2_(dba)_3_·CHCl_3_ [34] were prepared according to the literature. The other chemicals were obtained from commercial sources and used without purification.

### 4.2. Direct Arylation Polymerization of **1**-**Br** and **1**-**H**

A typical procedure is reported for run 3 in Table 1. The compounds Cs_2_CO_3_ (195 mg, 0.60 mmol) and pivalic acid (20.4 mg, 0.20 mmol) were placed in a 10 mL Schlenk tube equipped with a Teflon screw cock in a glove box. Then, **1**-**Br** (219 mg, 0.20 mmol), **2**-**H** (38.5 mg, 0.20 mmol), Pd_2_(dba)_3_·CHCl_3_ (1.0 mg, 1.0 μmol), **L1** (1.4 mg, 4.0 μmol), and 2-MeTHF (0.4 mL) were added. The mixture was stirred at room temperature for 0.5 h, and next at 100 °C for 24 h. The mixture was cooled up to room temperature, diluted with CHCl_3_ (100 mL), and washed with water (3 × 10 mL). The organic phase was poured into a vigorously stirred MeOH (400 mL). A dark red precipitate was collected by filtration, washed with MeOH, and dried under vacuum overnight. The product was subjected to Soxhlet extraction with acetone and hexane, and next with *o*-Cl_2_C_6_H_4_ (100 mL) containing diethylammonium diethyldithiocarbamate (10 mg). No colored substance remained in the extraction thimble. The *o*-Cl_2_C_6_H_4_ extract was poured into a vigorously stirred MeOH (400 mL), to produce a dark red solid of poly(**1**-*alt*-**2**), in >99% yield (231 mg, *M*_n_ = 22,700, *M*_w_/*M*_n_ = 3.9).

^1^H-NMR (C_2_D_2_Cl_4_, 130 °C, 600 MHz): δ 9.26 (d, *J* = 8.1 Hz, 2H), 7.35 (d, *J* = 3.3 Hz, 2H), 7.32 (d, *J* = 8.5 Hz, 2H), 7.14–7.08 (m, 4H), 7.04 (s, 2H), 3.77 (br, 4H), 2.11–2.01 (br, 2H), 1.54–1.22 (m, 80H), 0.94–0.86 (m, 12H).

## Figures and Tables

**Table 1:** DArP of **1-Br** and **2-H**.

Run ^1^	Solvent	Time (h)	Yield (%) ^2^	*M*_n_^3^	*M*_w_/*M*_n_^3^
1	toluene	5	99	17,000	2.6
2	THF	48	80	7,400	2.0
3	2-MeTHF	24	>99	22,700	3.9
3a	-	-	(63) ^4^	(44,900) ^4^	(2.2) ^4^
4 ^5^	toluene	24	93	15,700	2.3
5 ^5^	2-MeTHF	96	>99	14,100	2.5

^1^ Reactions were run at 100 °C using **1-Br** (0.20 mmol) and **2-H** (0.20 mmol), Pd_2_(dba)_3_·CHCl_3_ (0.5 mol%), **L1** (2 mol%), Cs_2_CO_3_ (0.60 mmol), and pivalic acid (0.20 mmol) in toluene (0.40 mL) unless otherwise stated. ^2^ Isolated yield after Soxhlet extraction with hexane, acetone, and *o*-Cl_2_C_6_H_4_. ^3^ Determined by GPC calibration based on polystyrene standards (140 °C, *o*-Cl_2_C_6_H_4_). ^4^ After removal of a low molecular weight portion from the product in run 3 by Soxhlet extraction with CHCl_3_. ^5^ The reaction was carried out in the presence of TMEDA (10 mol%).

**Table 2:** Electronic properties of poly(**1**-*alt*-**2**).

Entry	Method	*M*_n_ (PDI) ^1^	λ_max_^2^	λ_onset_^2^	*E*_g_^(opt/CV) 3^	*E*_ox_^4^	*E*_HOMO_^5^	*E*_red_^4^	*E*_LUMO_^5^
1	DArP	15,700 (2.3)	637, 699	747	1.66/1.73	0.53	−5.33	−1.20	−3.60
2	DArP	17,000 (2.6)	638, 700	746	1.66/1.74	0.54	−5.34	−1.20	−3.60
3	DArP	22,700 (3.9)	638, 700	747	1.66/1.75	0.54	−5.34	−1.21	−3.59
4	DArP	44,900 (2.2)	639, 701	753	1.65/1.74	0.54	−5.34	−1.20	−3.60
5	Stille	17,700 (1.7)	636, 699	748	1.66/1.76	0.54	−5.34	−1.22	−3.58
6	Stille	53,800 (2.8)	641, 702	747	1.66/1.73	0.56	−5.36	−1.17	−3.63

^1^ Determined by GPC calibration based on polystyrene standards (entries 1–5: 140 °C, *o*-Cl_2_C_6_H_4_; entry 6: 150 °C, 1,2,4-Cl_3_C_6_H_3_). ^2^ Values in nm; observed by UV-vis spectroscopy in thin films spin-coated on quartz plates. ^3^ Values in eV, estimated from the absorption onset (*E*_g_^opt^ = 1240/λ_onset_) and cyclic voltammetry (*E*_g_^CV^). ^4^ Onset potentials in V (vs. Fc/Fc^+^) for oxidation (*E*_ox_) and reduction (*E*_red_). ^5^ Values in eV, according to the following equation: *E*_HOMO_ = −4.80 − *E*_ox_; *E*_LUMO_ = −4.80 − *E*_red_.

**Table 3:** OFET characteristics and ordering parameters for poly(**1**-*alt*-**2**).

				Transistor Properties			Distance (Å) ^5^	
Entry	Method	M_n_^1^	M_w_^1^	μ_h_ (cm^2^ V^−1^ s^−1^) ^2^	V_th_ (V) ^3^	I_on_/I_off_^4^	d_l_^6^	d_π_^7^
1	DArP	15,700	35,800	0.31 (0.21–0.37)	−16 to −5	10^2^–10^8^	21.1	3.76
2	DArP	17,000	44,000	0.020 (0.013–0.030)	−15 to −5	10^2^–10^8^	22.0	3.76
3	DArP	22,700	88,200	0.016 (0.012–0.022)	−21 to −2	10^3^–10^6^	20.7	3.74
4	DArP	44,900	97,000	0.0030 (0.0024–0.0033)	−29 to −4	10^4^–10^7^	22.0	3.74
5	Stille	17,700	30,300	0.28 (0.15–0.51)	−29 to −3	10^2^–10^7^	22.0	3.79
6	Stille	53,800 ^8^	151,000 ^8^	0.004 (–) ^9^	−6	>10^5^	22.0	3.72

^1^ Determined by GPC calibration based on polystyrene standards (140 °C, *o*-Cl_2_C_6_H_4_), unless otherwise stated. ^2^ Average mobilities for over 5 devices tested unless otherwise stated. ^3^ The threshold voltage. ^4^ The on/off current ratio. ^5^ Determined by GIWAXS analysis. ^6^ *d*-Spacing to the lamellar structure of the edge-on crystallite, (100) along the *q*_z_ axis. ^7^ *d*-Spacing to the π–π stacking of the edge-on crystallites, (010) along the *q*_xy_ axis. ^8^ Determined by GPC calibration and based on polystyrene standards (150 °C, 1,2,4-Cl_3_C_6_H_3_). ^9^ Only one device showed FET characteristics among the 12 devices fabricated.

## Supplementary Materials

Supplementary materials including detailed experimental procedures, characterizations of all new compounds and polymers, as well as additional figures and tables are available online.
