# Supplementary material for: Synthesis of a 1,2-Dithienylethene-Containing Donor-Acceptor Polymer via Palladium-Catalyzed Direct Arylation Polymerization (DArP)
Source: Molecules. 2018 Apr 23;23(4):981. doi: 10.3390/molecules23040981 (PMC6017491; doi:10.3390/molecules23040981)
Supplement: Supplementary file 1 [file molecules-23-00981-s001.pdf]

Supplementary Material for

Synthesis of a 1,2-Dithienylethene-Containing Donor-Acceptor Polymer via Palladium-Catalyzed Direct Arylation Polymerization (DArP)

Masayuki Wakioka [1,*], Natsumi Yamashita [1], Hiroki Mori [2], Yasushi Nishihara [2] and Fumiyuki Ozawa [1,*]

[1] International Research Center for Elements Science (IRCELS), Institute for Chemical Research, Kyoto University, Uji, Kyoto 611-0011, Japan; yamashita.natsumi.22v@st.kyoto-u.ac.jp
[2] Research Institute for Interdisciplinary Science, Okayama University, 3-1-1 Tsushimanaka, Kita-ku, Okayama 700-8530, Japan; h-mor@okayama-u.ac.jp (H.M.); ynishiha@okayama-u.ac.jp (Y.N.)
* Correspondence: wakioka@scl.kyoto-u.ac.jp (M.W.); ozawa@scl.kyoto-u.ac.jp (F.O.)

Contents page

General Considerations S2
Migita–Kosugi–Stille Cross-coupling Polymerization of 1-Br and 2-SnMe$_3$ S2
Fabrication of OFET Devices S3
Effects of the Solvent for Spin-coating on Transistor Properties (Table S1) S3
^{1}H NMR Assignments of Polymers S4
Synthesis of Reference Compounds for ^{1}H NMR Assignments S5
1. Synthesis of **Br-1-2-1-Br** S5
2. Synthesis of **H-1-2-1-H** S5
3. Synthesis of **H-2-1-2-H** S6
4. Synthesis of **1$_3$-H** S6
5. Synthesis of **2$_2$-H** and Poly(**1**-*alt*-**2$_2$**) S7
References S9

Figure S1. Cyclic voltammograms of poly(**1**-*alt*-**2**) in thin films. S9
Figure S2. Transfer characteristics of the OFETs based on poly(**1**-*alt*-**2**). S10
Figure S3. Output characteristics of the OFETs based on poly(**1**-*alt*-**2**). S10
Figure S4. GIWAXS images of the thin films of poly(**1**-*alt*-**2**). S11
Figure S5. In-plane and out-of-plane line-cuts of GIWAXS measurement for the thin films of poly(**1**-*alt*-**2**). S11
Figure S6. AFM images of the thin films of poly(**1**-*alt*-**2**). S12
Figure S7. ^{1}H NMR spectrum of poly(**1**-*alt*-**2**) prepared via DArP (M_n = 15,700). S12
Figure S8. ^{1}H NMR spectrum of poly(**1**-*alt*-**2**) prepared via DArP (M_n = 17,000). S13
Figure S9. ^{1}H NMR spectrum of poly(**1**-*alt*-**2**) prepared via DArP (M_n = 22,700). S13
Figure S10. ^{1}H NMR spectrum of poly(**1**-*alt*-**2**) prepared via Migita–Kosugi–Stille cross-coupling polymerization (M_n = 17,700). S14
Figure S11. ^{1}H NMR spectrum of poly(**1**-*alt*-**2**) prepared via Migita–Kosugi–Stille cross-coupling polymerization (M_n = 24,400). S14
Figure S12. ^{1}H NMR spectrum of poly(**1**-*alt*-**2$_2$**). S15

General Considerations

NMR spectra were recorded on 400 MHz (^{1}H NMR 400.13 MHz and ^{13}C NMR 150.62 MHz) and 600 MHz (^{1}H NMR 600.13 MHz and ^{13}C NMR 150.92 MHz) (Bruker). Chemical shifts are reported in δ (ppm), referenced to ^{1}H (residual) and ^{13}C signals of deuterated solvents as internal standards. Analytical GPC was performed on a HLC 8120 GPC system with a TSKgel GMHHR-H(S)HT column (mobile phase: o-$Cl_2C_6H_4$, flow rate: 1.0 mL min^{-1}, temperature: 140 °C), a JASCO GPC assembly consisting of a model PU-980 precision pump, a model RI-1530 refractive index detector, and three polystyrene gel columns (Shodex KF-801, KF-803L, KF-805L, mobile phase: $CHCl_3$, flow rate: 1.0 mL min^{-1}, temperature: 40 °C), or PL-220 GPC system with HT-806M column (mobile phase: 1,2,4-$Cl_3C_6H_3$, flow rate: 1.0 mL min^{-1}, temperature: 150 °C). The columns were calibrated against 10 (M_n = 1,200–2,610,000 for TSKgel GMHHR-H(S)HT column), 9 (M_n = 1,220–2,700,000) for Shodex KF-801, KF-803L, KF-805L), or 14 standard polystyrene samples (M_n = 182–8,440,000 for HT-806M column). Recycling preparative GPC was performed on a JAI LC918U instrument equipped with JAIGEL-1H-40 and -2H-40 columns. Chloroform was used as the mobile phase with a flow rate 15 mL min^{-1}. High-resolution mass spectra were recorded on a Bruker micrOTOF I spectrometer in the atmospheric-pressure chemical ionization (APCI) mode. UV-vis absorption was recorded on a JASCO V-560 spectrometer. Cyclic voltammograms (CVs) were recorded on electrochemical analyzer ALS600E in CH_3CN containing Bu_4NPF_6 (0.1 M) as supporting electrolyte at a scan rate of 100 mV/s. A glassy carbon electrode (ϕ = 3 mm), an Ag/Ag^+ (Ag wire in 0.01 M $AgNO_3$/0.1 M Bu_4NPF_6/CH_3CN), and a Pt wire electrode were used as working, reference, and counter electrodes, respectively. Samples of the polymer films were prepared by dropcasting on a working electrode from their o-$Cl_2C_6H_4$ solutions. All the potentials were calibrated with the half-wave potential of the ferrocene/ferrocenium redox couple measured under identical condition (Fc/Fc^+: $E_{1/2}$ = +0.08 V measured under identical conditions). The AFM images of the SAMs were measured on a NanoNavi IIs Probe Station equipped with a probing microscope unit, Nanocute (Seiko Instruments), and the probe unit was put on an antivibration stage. The dynamic (tapping) mode was used for the AFM scanning. The cantilever was made of crystalline silicon tip, and its force constant was 40 N m^{-1}. GIWAXS experiments were conducted at the SPring-8 on beamline BL46XU. The sample was irradiated at a fixed incident angle on the order of 0.12° through a Huber diffractometer with an X-ray energy of 12.39 keV (λ = 1 Å), and the GIWAXS patterns were recorded with a 2-D image detector (Pilatus 300K). Samples for the X-ray measurements were prepared by spin-casting the polymer on the OTS-modified Si/SiO_2. Elemental analysis was performed by ICR Analytical Laboratory, Kyoto University. 1-Bromo-2-decyltetradecane [1], **3** [2], **5** [3], $[Ir(OMe)(cod)]_2$ [4], and $Pd(dba)_2$ [5] were prepared according to the literature.

Migita–Kosugi–Stille Cross-coupling Polymerization of 1-Br and 2-SnMe$_3$

A mixture of **1-Br** (219 mg, 0.20 mmol), **2-SnMe$_3$** (104 mg, 0.20 mmol), $Pd_2(dba)_3{\cdot}CHCl_3$ (4.2 mg, 4.0 μmol) and $P(2\text{-}MeC_6H_4)_3$ (9.7 mg, 32 μmol) in toluene (20 mL) was heated at 115 °C for 48 h. The mixture was cooled to room temperature, and poured into vigorously stirred MeOH (400 mL). A dark red precipitate formed was collected by filtration, washed with MeOH, and dried under vacuum overnight. The product was subjected to Soxhlet extraction with acetone and hexane, and then with o-$Cl_2C_6H_4$ (100 mL) containing diethylammonium diethyldithiocarbamate (10 mg). No colored material remained in the extraction thimble. The o-$Cl_2C_6H_4$ extract was poured into vigorously stirred MeOH (400 mL), to give a dark red solid of poly(**1**-*alt*-**2**) in 95% yield (214 mg). Because a part of the polymer was insoluble at 140 °C in o-$Cl_2C_6H_4$, the analysis by high-temperature GPC using o-$Cl_2C_6H_4$ at 140 °C was infeasible. Instead, the M_n could be estimated by GPC using 1,2,4-$Cl_3C_6H_3$ at 145 °C (M_n = 53,800, PDI = 2.8). A part of the product (50.0 mg) was subjected to Soxhlet extraction with $CHCl_3$. The extract was concentrated (ca. 30 mL), and poured into vigorously stirred MeOH (200 mL) to give poly(**1**-*alt*-**2**) (9.0 mg, 18% recovery, M_n = 24,400, M_w/M_n = 2.5).

The reaction of **1-Br** and **2-SnMe$_3$** (each 0.20 mmol) was similarly conducted using $Pd_2(dba)_3 \cdot CHCl_3$ (2.1 mg, 2.0 μmol), $P(2\text{-}MeC_6H_4)_3$ (4.9 mg, 16 μmol), and toluene (25 mL) at 90 °C for 24 h. The mixture was cooled to room temperature, and poured into vigorously stirred MeOH/H_2O (180/120 mL). A dark red precipitate formed was collected by filtration, washed with MeOH, and dried under vacuum overnight. The product was subjected to Soxhlet extraction with $CHCl_3$ (120 mL) containing diethylammonium diethyldithiocarbamate (22 mg). The extract was concentrated (ca. 20 mL), and poured into vigorously stirred MeOH (200 mL) to give poly(**1**-*alt*-**2**) in >99% yield (234 mg, M_n = 17,700, PDI = 1.7).

Fabrication of OFET Devices

Typical bottom-gate top-contact OFET devices were fabricated as follows. All processes, except for substrate cleaning, were performed under a nitrogen atmosphere. A heavily doped *n*-Si wafer with 200 nm thick thermally grown SiO_2 (C_i = 17.3 nF cm^{-2}) as the dielectric layer was used as the substrate. The n^+-Si/SiO_2 substrates were carefully cleaned by ultrasonication with acetone and isopropanol for 10 min, respectively. After drying, the substrates were irradiated with UV–O_3 for 20 min and then treated with a vapor of *n*-octyltriethoxysilane (OTS) to form an SAM. Polymer layers were then spin-coated from solution in $CHCl_3$ (4 g/L for $CHCl_3$ soluble polymers: entries 1 and 5 in Table 2) at 3000 rpm for 30 s or solution in *o*-$Cl_2C_6H_4$ (4 g/L for $CHCl_3$ insoluble polymers: entries 3–4, 6 in Table 2) at 1000 rpm for 10 s and then 2500 rpm for 35 s, and subsequently annealed at 240 °C for 30 min on the hotplate in the glovebox. On top of the polymer thin films, Au drain and source electrodes (thickness 80 nm) were deposited under reduced pressure (5×10^{-5} Pa) through a shadow mask, where drain–source channel length (L) and width (W) are 100 μm and 2000 μm, respectively. The current–voltage characteristics of the OFET devices were measured at room temperature in air on a Keithley 6430 sub-femto ampere remote sourcemeter combined with a Keithley 2400 measure-source unit. Field-effect mobilities were calculated in the saturation regime of I_D using the following equation: $I_D = (WC_i/2L)\mu(V_G - V_{th})^2$, where C_i is the capacitance of the SiO_2 insulator; I_D is the source–drain current; and V_D, V_G, and V_{th} are the source–drain, gate, and threshold voltages, respectively. The current on/off ratio (I_{on}/I_{off}) was determined from a minimum I_D at around V_G = –60 V.

Effects of the Solvent for Spin-coating on Transistor Properties

In a preliminary experiments, we checked the effects of the solvent for spin-coating on transistor properties using poly(**1**-*alt*-**2**) prepared via DArP (M_n = 15,700; run 5 in Table 1, entry 1 in Table 2). The OFET devices were fabricated as shown above except for annealing conditions (at 230 °C for 10 min). Table S1 summarizes the OFET characteristics. The results showed that the choice of the solvent ($CHCl_3$ or *o*-$Cl_2C_6H_4$) for the spin-coating of poly(**1**-*alt*-**2**) little affected on the carrier mobility.

Table S1. OFET characteristics of poly(**1**-*alt*-**2**).

entry	solvent	μ_h, max (cm^2 V^{-1} s^{-1}) [1]	μ_h, average (cm^2 V^{-1} s^{-1}) [1]	V_{th} [2] (V)	I_{on}/I_{off} [3]
1	$CHCl_3$	0.27	0.19	–19	10^5–10^7
2	*o*-$Cl_2C_6H_4$	0.20	0.12	–14	10^3–10^5

[1] Calculated in the saturation regimes. [2] Threshold voltage. [3] On-off current ratio.

^{1}H NMR Assignments of Polymers

The ^{1}H NMR signals of poly(**1**-*alt*-**2**) were assigned referring to the following compounds. The chemical shifts were observed in $C_2D_2Cl_4$ at 130 °C.

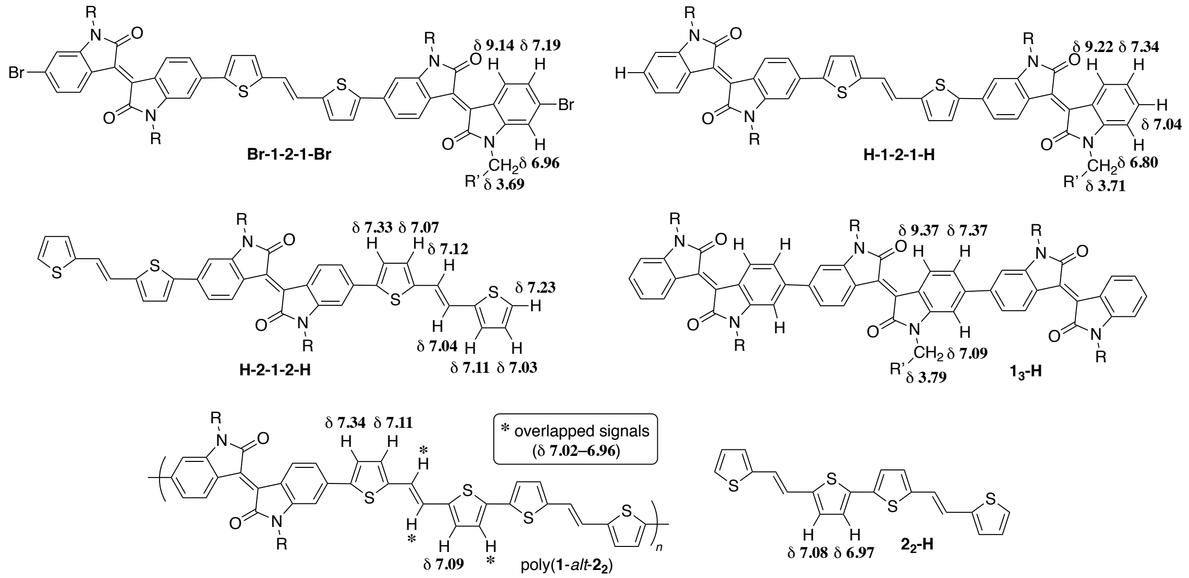

The chemical shifts of poly(**1**-*alt*-**2**) are as follows:

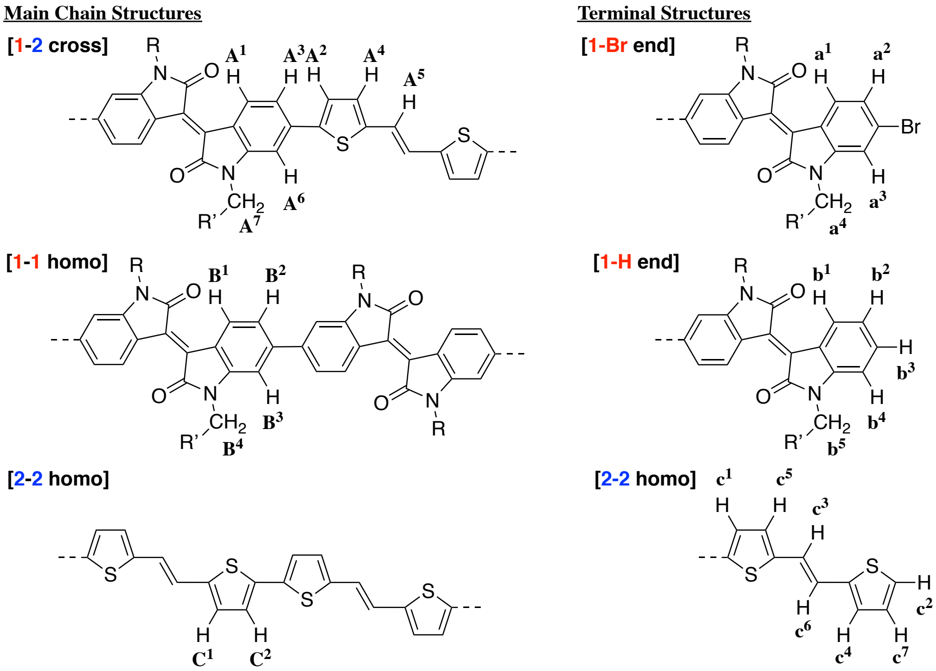

Main chain: δ 9.26 (**A**[1]), 7.35 (**A**[2]), 7.32 (**A**[3]), 7.12 (**A**[4]), 7.11 (**A**[5]), 7.04 (**A**[6]), 3.77 (**A**[7]).
δ 9.39 (**B**[1]) (**B**[2]–**B**[4] and **C**[1]–**C**[2] were not observed.)

Terminal: δ 9.14 (**a**[1]), 7.19 (**a**[2]), 6.96 (**a**[3]), 3.69 (**a**[4]).
δ 9.22 (**b**[1]), 6.80 (**b**[4]), 3.71 (**b**[5]) (**b**[2] and **b**[3] were overlapped with other signals).
δ 7.23 (**c**[2]), 7.12 (**c**[3]), 7.08 (**c**[5]), 7.04 (**c**[6]) (**c**[1], **c**[4], and **c**[7] were overlapped with other signals).

Synthesis of Reference Compounds for ^{1}H NMR Assignments

The synthetic procedures for the other compounds are as follows.

1. Synthesis of ***Br-1-2-1-Br***

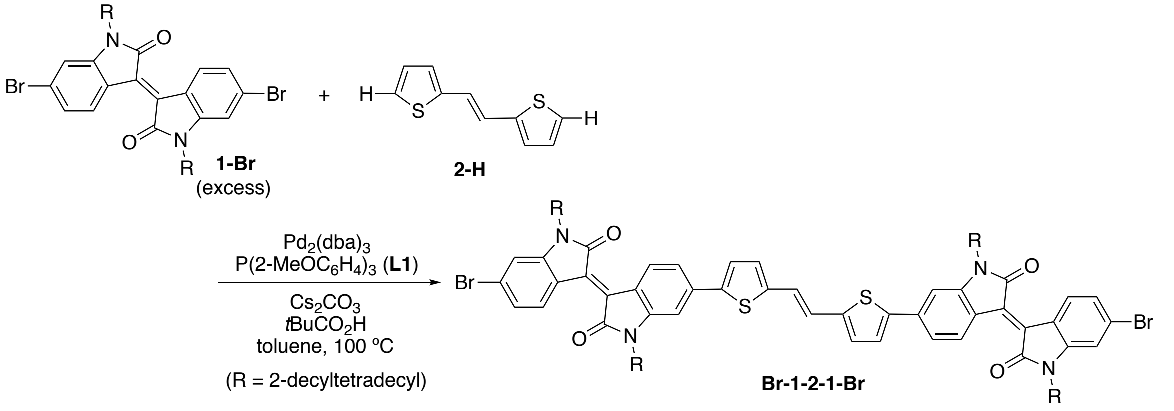

The compounds Cs_2CO_3 (48.9 mg, 0.15 mmol) and pivalic acid (5.1 mg, 0.050 mmol) were placed in a 10 mL Schlenk tube in a glovebox, and **1-Br** (219 mg, 0.20 mmol), **2-H** (9.6 mg, 0.050 mmol), $Pd_2(dba)_3 \cdot CHCl_3$ (1.0 mg, 1.0 μmol), **L1** (1.4 mg, 4.0 μmol), and toluene (0.4 mL) were added. The mixture was stirred at room temperature for 0.5 h, and then at 100 °C for 115 h. The reaction mixture was cooled to room temperature, diluted with $CHCl_3$ (5 mL), and washed with water (3 × 2 mL). The organic phase was separated and concentrated to dryness under reduced pressure. The residue was subjected to flash column chromatography (SiO_2, CH_2Cl_2, and then CH_2Cl_2/AcOEt = 1/1). The crude product was purified by recycle GPC to afford the title compound as a black solid (85.5 mg, 77% yield). ^{1}H NMR ($C_2D_2Cl_4$, 130 °C, 400 MHz): δ 9.24 (d, *J* = 8.5 Hz, 2H), 9.12 (d, *J* = 8.6 Hz, 2H), 7.35 (d, *J* = 3.8 Hz, 2H), 7.31 (dd, *J* = 8.5, 1.8 Hz, 2H), 7.19 (dd, *J* = 8.6, 1.7 Hz, 2H), 7.11 (d, *J* = 3.8 Hz, 2H), 7.11 (d, *J* = 1.8 Hz, 2H), 7.03 (s, 2H), 6.96 (d, *J* = 1.7 Hz, 2H), 3.75 (d, *J* = 6.7 Hz, 4H), 3.69 (d, *J* = 6.9 Hz, 4H) 2.10–1.93 (br, 4H), 1.54–1.18 (m, 160H), 0.97–0.83 (m, 24H). HRMS: *m*/*z* calcd for $C_{138}H_{214}Br_2N_4O_4S_2$ 2214.4546 ($[M + 1]^+$), found 2214.4733.

2. Synthesis of ***H-1-2-1-H***

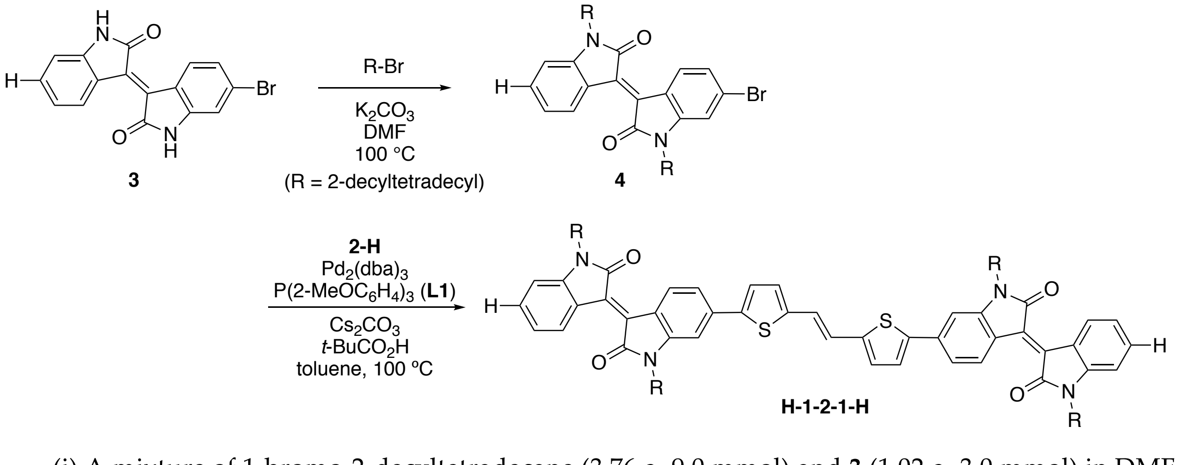

(i) A mixture of 1-bromo-2-decyltetradecane (3.76 g, 9.0 mmol) and **3** (1.02 g, 3.0 mmol) in DMF (20 mL) was stirred at 100 °C overnight. After cooling to room temperature, water (100 mL) was added, and the mixture was extracted with Et_2O (3 × 100 mL). The combined extracts were washed with brine (50 mL), dried over $MgSO_4$, and evaporated under reduced pressure. The residue was passed through a short column (SiO_2, hexane/CH_2Cl_2 = 1/1). The crude product was purified by flash column chromatography (SiO_2) using MeOH as an eluent to remove a yellow oily material. Then, CH_2Cl_2 was used as an eluent, affording the product contaminated with SiO_2. Further purification by flash column chromatography (SiO_2, hexane/CH_2Cl_2 = 5/1) gave **4** as a dark red solid

(719 mg, 24% yield). ^{1}H NMR ($CDCl_3$, 25 °C, 400 MHz): δ 9.16 (dd, *J* = 8.2, 1.0 Hz, 1H), 9.09 (d, *J* = 8.6 Hz, 1H), 7.35 (td, *J* = 7.8, 1.1 Hz, 1H), 7.16 (dd, *J* = 8.6, 1.8 Hz, 1H), 7.04 (td, *J* = 7.8, 1.0 Hz, 1H), 6.90 (d, *J* =1.8 Hz, 1H), 6.77 (d, *J* = 7.8 Hz, 1H), 3.67–3.62 (m, 4H), 1.94–1.86 (m, 2H), 1.43–1.20 (m, 80H), 0.91–0.84 (m, 12H). ^{13}C{^{1}H} NMR ($CDCl_3$, 25 °C, 100 MHz): δ 168.3, 168.1, 146.2, 145.4, 134.0, 132.6, 132.1, 131.1, 130.1, 126.3, 126.3, 125.0, 122.3, 121.7, 120.6, 111.4, 108.2, 44.7, 44.7, 36.3, 36.2, 32.1, 32.1, 31.8, 31.6, 30.1, 29.8, 29.8, 29.8, 29.8, 29.5, 29.5, 26.6, 26.5, 22.8, 14.2 (each s). HRMS: *m*/*z* calcd for $C_{64}H_{105}N_2O_2$ 1013.7432 ($[M + 1]^+$), found 1013.7465.

(ii) The compounds Cs_2CO_3 (196 mg, 0.60 mmol) and pivalic acid (20.4 mg, 0.20 mmol) were placed in a 10 mL Schlenk tube in a glovebox, and **4** (203 mg, 0.20 mmol), **2-H** (19.2 mg, 0.10 mmol), $Pd_2(dba)_3{\cdot}CHCl_3$ (1.0 mg, 1.0 μmol), **L1** (1.4 mg, 4.0 μmol), and toluene (0.4 mL) were added. The mixture was stirred at room temperature for 0.5 h, and then at 100 °C for 48 h. The reaction mixture was cooled to room temperature, diluted with $CHCl_3$ (5 mL), and washed with water (3 × 2 mL). The organic phase was separated and concentrated to dryness under reduced pressure. The residue was subjected to flash column chromatography (SiO_2, CH_2Cl_2, and then CH_2Cl_2/AcOEt = 1/1). The crude product was purified by recycle GPC to afford the title compound as a black solid (100 mg, 49% yield). ^{1}H NMR ($C_2D_2Cl_4$, 130 °C, 400 MHz): δ 9.27 (d, *J* = 8.2 Hz, 2H), 9.22 (d, *J* = 7.6 Hz, 2H), 7.34 (t, *J* = 7.6 Hz, 2H), 7.34 (d, *J* = 3.4 Hz, 2H), 7.33–7.28 (m, 2H), 7.12–7.09 (m, 2H), 7.10 (s, 2H), 7.04 (t, *J* = 7.6 Hz, 2H), 7.03 (s, 2H), 6.80 (d, *J* = 7.6 Hz, 2H), 3.76 (d, *J* = 7.6 Hz, 4H), 3.71 (d, *J* = 7.2 Hz, 4H), 2.10–1.95 (br, 4H), 1.53–1.21 (m, 160H), 0.96–0.84 (m, 24H). HRMS: *m*/*z* calcd for $C_{138}H_{216}N_4O_4S_2$ 2058.6336 ($[M + 1]^+$), found 2058.6374.

3. *Synthesis of **H-2-1-2-H***

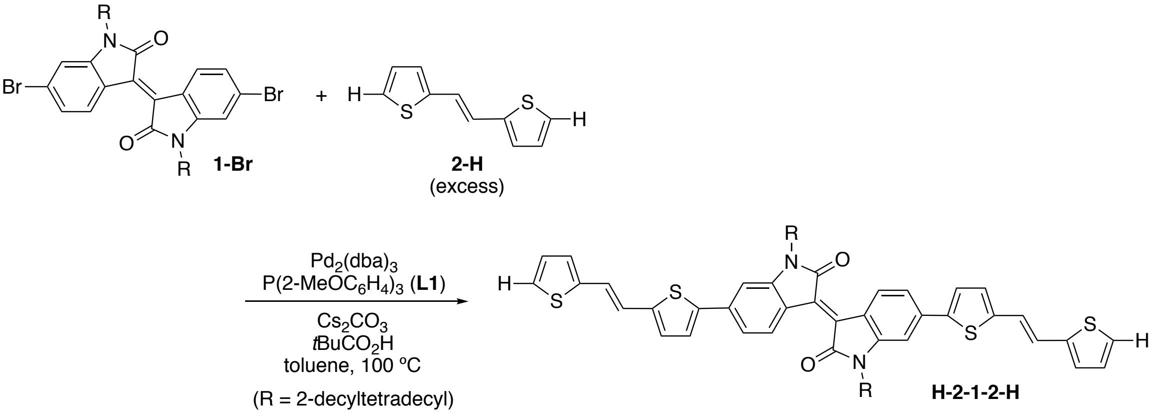

The compounds Cs_2CO_3 (48.9 mg, 0.15 mmol) and pivalic acid (5.1 mg, 0.050 mmol) were placed in a 10 mL Schlenk tube in a glovebox, and **1-Br** (54.7 mg, 0.050 mmol), **2-H** (38.5 mg, 0.20 mmol), $Pd_2(dba)_3{\cdot}CHCl_3$ (1.0 mg, 1.0 μmol), **L1** (1.4 mg, 4.0 μmol), and toluene (0.4 mL) were added. The mixture was stirred at room temperature for 0.5 h, and then at 100 °C for 115 h. The reaction mixture was cooled to room temperature, diluted with $CHCl_3$ (5 mL), and washed with water (3 × 2 mL). The organic phase was separated and concentrated to dryness under reduced pressure. The residue was subjected to flash column chromatography (SiO_2, CH_2Cl_2, and then CH_2Cl_2/AcOEt = 1/1). The crude product was purified by recycle GPC to afford the title compound as a black solid (29.8 mg, 45% yield). ^{1}H NMR ($C_2D_2Cl_4$, 130 °C, 400 MHz): δ 9.25 (d, *J* = 8.4 Hz, 2H), 7.33 (d, *J* = 3.8 Hz, 2H), 7.30 (dd, *J* = 8.4, 1.8 Hz, 2H), 7.23 (d, *J* = 5.1 Hz, 2H), 7.12 (d, *J* = 15.9 Hz, 2H), 7.11 (d, *J* = 3.7 Hz, 2H), 7.07 (d, *J* = 3.8 Hz, 2H), 7.04 (d, *J* = 15.9 Hz, 2H), 7.03 (d, *J* = 1.8 Hz, 2H), 7.03 (d, *J* = 5.1, 3.7 Hz, 2H), 3.76 (d, *J* = 7.2 Hz, 4H), 2.10–2.00 (br, 2H), 1.55–1.22 (m, 80H), 0.96–0.84 (m, 12H). HRMS: *m*/*z* calcd for $C_{84}H_{118}N_2O_2S_4$ 1315.8149 ($[M + 1]^+$), found 1315.8201.

4. *Synthesis of **1$_3$-H***

This compound was prepared by a two-step procedure as outlined below.

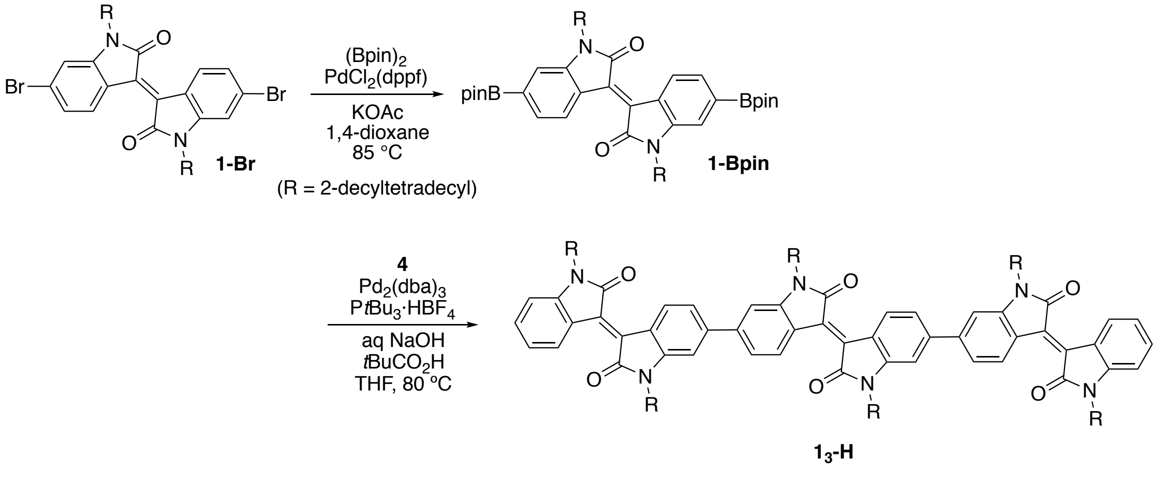

(i) A mixture of **1-Br** (3.28 g, 3.0 mmol), bis(pinacolato)diboron (1.83 g, 7.2 mmol), [$PdCl_2$(dppf)] (132 mg, 0.18 mmol), potassium acetate (1.77 g, 18 mmol), and 1,4-dioxane (10 mL) was stirred at 80 °C overnight. The solvent was removed under reduced pressure, and the residue was subjected to a short column (SiO_2, CH_2Cl_2). The crude product was purified by flash column chromatography (SiO_2, hexane/AcOEt = 50/1, and then 20/1), and subsequently recrystallized from CH_2Cl_2/MeOH at –20 °C to afford **1-Bpin** as a dark red solid (1.04 g, 29% yield). ^{1}H NMR ($CDCl_3$, 25 °C, 400 MHz): δ 9.13 (d, J = 7.9 Hz, 2H), 7.47 (d, J = 7.9 Hz, 2H), 7.15 (s, 2H), 3.68 (d, J = 7.3 Hz, 4H), 2.00–1.90 (br, 2H), 1.64–1.16 (m, 80H), 1.36 (s, 24H), 0.90–0.83 (m, 12H).

(ii) To a solution of **4** (101 mg, 0.10 mmol), **1-Bpin** (59.0 mg, 0.050 mmol), $Pd_2(dba)_3{\cdot}CHCl_3$ (0.50 mg, 0.50 μmol), P*t*$Bu_3{\cdot}HBF_4$ (0.60 mg, 2.0 μmol) in THF (0.5 mL) were added 3.0 M aqueous NaOH (50 μL, 0.15 mmol). The mixture was stirred at 80 °C for 1.5 h. The mixture was cooled to room temperature, diluted with CH_2Cl_2 (5 mL), and washed with water (2 × 5 mL). The organic phase was passed through a short column (SiO_2, CH_2Cl_2), and concentrated to dryness. The residue was purified by recycle GPC, and the crude product was dissolved in a minimum amount of $CHCl_3$ (ca. 1 mL). The solution poured into vigorously stirred acetone (75 mL), and a black solid thus precipitated was collected by filtration, washed with MeOH, and dried under vacuum overnight (52.0 mg, 37% yield). ^{1}H NMR ($CDCl_3$, 25 °C, 400 MHz): δ 9.29 (d, J = 8.2 Hz, 2H), 9.27 (d, J = 8.2 Hz, 2H) 9.20 (d, J = 7.9 Hz, 2H), 7.34 (t, J = 7.7 Hz, 2H), 7.31 (d, J = 8.2 Hz, 4H), 7.05 (t, J = 7.8 Hz, 2H), 7.03–6.99 (br, 4H), 6.77 (d, J = 7.9 Hz, 2H), 3.83–3.72 (br, 8H), 3.72–3.64 (br, 4H), 2.06–1.86 (br, 6H), 1.52–1.14 (m, 240H), 0.92–0.80 (m, 36H). ^{1}H NMR ($C_2D_2Cl_4$, 130 °C, 400 MHz): δ 9.37 (d, J = 8.4 Hz, 2H), 9.35 (d, J = 8.4 Hz, 2H), 9.24 (d, J = 8.0 Hz, 2H), 7.38–7.32 (m, 6H), 7.11–7.03 (m, 6H), 6.81 (d, J = 7.9 Hz, 2H), 3.82–3.75 (br, 8H), 3.74–3.68 (br, 4H), 2.12–1.96 (br, 6H), 1.55–1.20 (m, 240H), 0.97–0.83 (m, 36H). Anal. Calcd. for $C_{192}H_{314}N_6O_6$: C, 82.28; H, 11.29; N, 3.00. Found: C, 82.22; H, 11.30; N, 2.96.

5. Synthesis of ***2***$_2$***-H*** *and Poly(**1**-alt-**2**$_2$)*

This compound was prepared by a four-step procedure as outlined below.

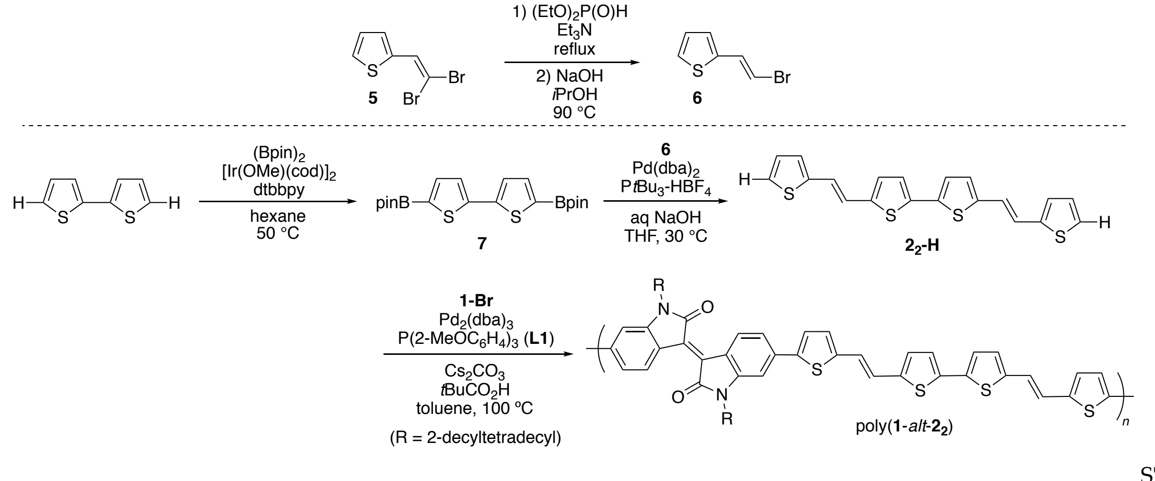

(i) A mixture of 2-(2,2-dibromoethenyl)thiophene (**5**; 4.46 g, 10 mmol), diethyl phosphite (15.0 g, 110 mmol), and Et_3N (25.4 mL, 180 mmol) was stirred under reflux for 3 h. The reaction mixture was cooled to room temperature, poured into 2 N aqueous HCl (200 mL), and extracted with Et_2O (3 × 100 mL). The combined extracts were washed with brine (100 mL), dried over $MgSO_4$, and concentrated under reduced pressure. The residue was passed through a short column (SiO_2, hexane), giving a mixture of geometric isomers of **6** (*E*/*Z* = 80/20, 6.47 g). Then, a mixture of the crude product (6.47 g) and NaOH (1.60g, 40 mmol) in 2-propanol (70 mL) was stirred at 90 °C for 1 h. After cooling to room temperature, water (200 mL) was added, and the mixture was extracted with Et_2O (3 × 100 mL). The combined extracts were washed with brine (100 mL), dried over $MgSO_4$, and concentrated under reduced pressure. The residue was passed through a short column (SiO_2, hexane), giving geometrically pure **6** as yellow oily material (4.81 g, 70%). The NMR data were identical to those reported [6]. ^1H NMR ($CDCl_3$, 25 °C, 400 MHz): δ 7.23–7.17 (m, 2H), 7.00–6.95 (m, 2H), 6.63 (d, *J* = 13.9 Hz, 1H).

(ii) A mixture of 2,2′-bithiophene (1.66 g, 10 mmol), bis(pinacolato)diboron (2.79 g, 11 mmol), $[Ir(OMe)(cod)]_2$ (133 mg, 0.20 mmol), 4,4′-di-*tert*-butyl-2,2′-bipyridyl (107 mg, 0.40 mmol), and hexane (50 mL) was stirred at 50 °C for 15 min. After cooling to room temperature, the volatiles were removed under reduced pressure. The residue was passed through a short column (SiO_2, hexane), and the crude product was subsequently recrystallized from CH_2Cl_2/MeOH at –20 °C to afford **7** as a colorless crystals (3.41 g, 82%). The NMR data were identical to those reported [7]. ^1H NMR ($CDCl_3$, 25 °C, 400 MHz): δ 7.52 (d, *J* = 3.6 Hz, 2H), 7.29 (d, *J* = 3.6 Hz, 2H), 1.35 (s, 24H).

(iii) To a solution of **6** (189 mg, 1.0 mmol), **7** (209 mg, 0.50 mmol), $Pd(dba)_2$ (11.5 mg, 20 μmol), $P\textit{t}Bu_3{\cdot}HBF_4$ (14.5 mg, 50 μmol) in THF (5 mL) were added 3.0 M aqueous NaOH (1.0 mL, 3.0 mmol). The mixture was stirred at 30 °C for 20 h. The mixture was diluted with $CHCl_3$ (20 mL), passed through a short column (SiO_2, CH_2Cl_2), and concentrated under reduced pressure. The residue was subjected to flash column chromatography (SiO_2, hexane/CH_2Cl_2 = 10/1, and then CH_2Cl_2), and subsequently recrystallized from $CHCl_3$/MeOH at –20 °C to afford $\mathbf{2_2}$**-H** as orange crystals (171 mg, 89%). ^1H NMR ($CDCl_3$, 25 °C, 400 MHz): δ 7.21 (d, *J* = 4.8 Hz, 2H), 7.08–7.03 (m, 4H), 7.02–6.98 (m, 6H), 6.94 (d, *J* = 3.8 Hz, 2H). ^1H NMR ($C_2D_2Cl_4$, 130 °C, 400 MHz): δ 7.21 (d, *J* = 4.7 Hz, 2H), 7.08 (d, *J* = 3.6 Hz, 4H), 7.05 (d, *J* = 16.2 Hz, 2H), 7.01 (dd, *J* = 4.7, 3.6 Hz, 2H), 6.99 (d, *J* = 16.2 Hz, 2H), 6.97 (d, *J* = 3.6 Hz, 2H). $^{13}C\{^1H\}$ NMR analysis was infeasible due to low solubility. Anal. Calcd. for $C_{20}H_{14}S_4$: C, 62.79; H, 3.69. Found: C, 62.75; H, 3.70.

(iv) The compounds Cs_2CO_3 (97.7 mg, 0.30 mmol) and pivalic acid (10.2 mg, 0.10 mmol) were placed in a 10 mL Schlenk tube in a glovebox, and **1-Br** (109 mg, 0.10 mmol), $\mathbf{2_2}$**-H** (38.3 mg, 0.10 mmol), $Pd_2(dba)_3{\cdot}CHCl_3$ (1.0 mg, 1.0 μmol), **L1** (1.4 mg, 4.0 μmol), and THF (0.4 mL) were added. The mixture was stirred at room temperature for 0.5 h, and then at 100 °C for 2 h. The reaction mixture was cooled to room temperature, diluted with $CHCl_3$ (100 mL), and washed with water (3 × 10 mL). The organic phase was poured into vigorously stirred MeOH (400 mL), and a dark red precipitate was collected by filtration, washed with MeOH, and dried under vacuum overnight. The product was subjected to Soxhlet extraction with acetone and $CHCl_3$, and then with PhCl. The PhCl extract was poured into vigorously stirred MeOH (400 mL), to give a dark red solid of poly(**1**-*alt*-$\mathbf{2_2}$) in 27% yield (35.8 mg, M_n = 10,500, M_w/M_n = 1.9). ^1H NMR ($C_2D_2Cl_4$, 130 °C, 600 MHz): δ 9.25 (d, *J* = 8.3 Hz, 2H), 7.34 (d, *J* = 2.0 Hz, 2H), 7.30 (d, *J* = 8.3 Hz, 2H), 7.11 (d, *J* = 3.4 Hz, 2H), 7.09 (d, *J* = 3.7 Hz, 2H), 7.06–7.03 (m, 2H), 7.02–6.96 (m, 6H), 3.77 (d, *J* = 5.9 Hz, 4H), 2.01–1.95 (br, 2H), 1.58–1.22 (m, 80H), 0.97–0.85 (m, 12H).

References

1. Fu, B.; Baltazar, J.; Sankar, A. R.; Chu, P.-H.; Zhang, S.; Collard, D. M.; Reichmanis, E. *Adv. Funct. Mater.* **2014**, *24*, 3734.
2. Mei, J.; Graham, K, R.; Stalder, R.; Reynolds, J. R. *Org. Lett.* **2010**, *12*, 660.
3. Rosiak, A.; Frey, W.; Christoffers, J. *Eur. J. Org. Chem.* **2006**, 4044.
4. Hurst, T. E.; Macklin, T. K.; Becker, M.; Hartmann, E.; Kuegel, W.; Parisienne-La S., J.-C.; Batsanov, A. S.; Marder, T. B.; Snieckus, V. *Chem. Eur. J.* **2010**, *16*, 8155.
5. Ukai, T.; Kawamura, H.; Ishii, Y. *J. Organomet. Chem.* **1974**, 65, 253.
6. Rossi, R.; Carpita, A.; Lippolis, V. *Synth. Commun.* **1991**, *21*, 333.
7. Chen, K.; Gallaher, J. K.; Barker, A. J.; Hodgkiss, J. M. *J. Phys. Chem. Lett.* **2014**, *5*, 1732.

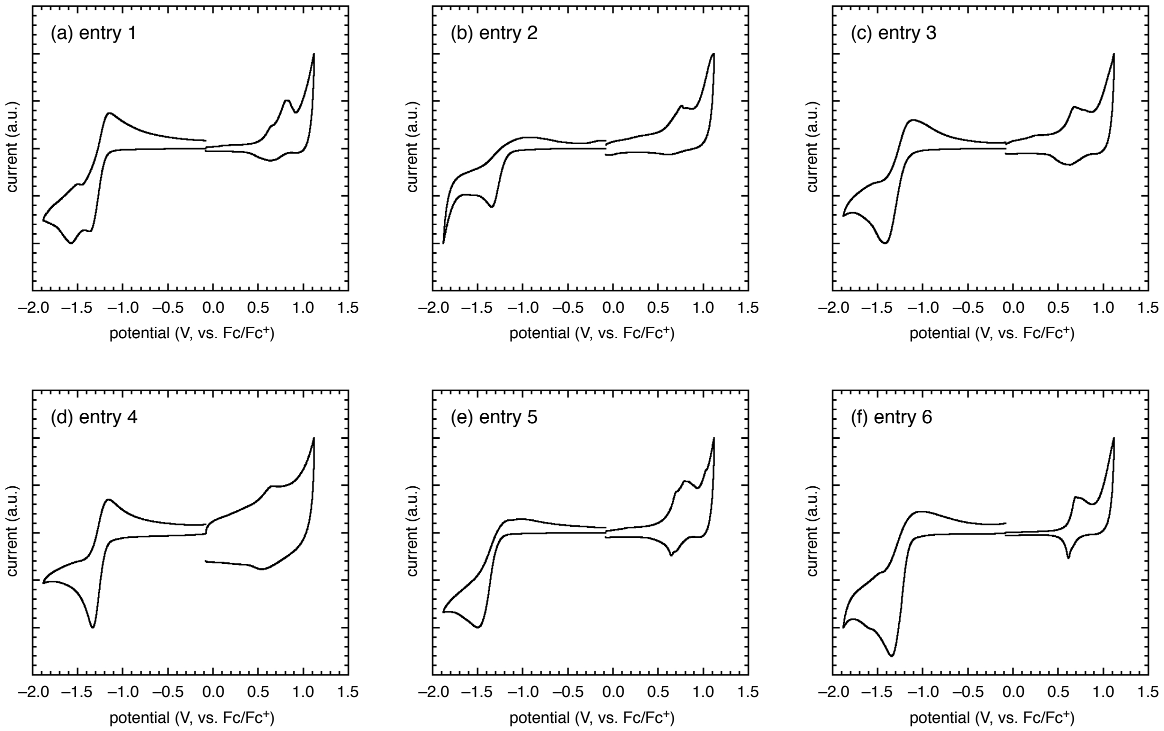

Figure S1. Cyclic voltammograms of poly(**1**-*alt*-**2**) of entries 1–6 in thin film. The entry number follows Table 2.

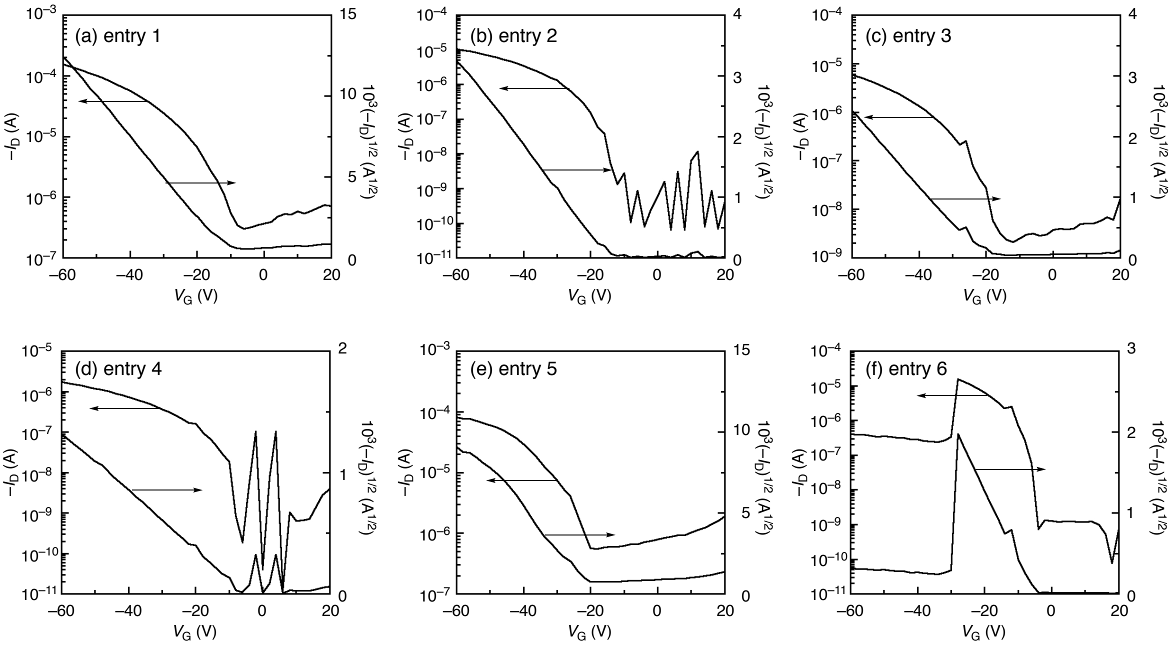

Figure S2. Transfer characteristics of the OFETs based on poly(**1**-*alt*-**2**) of entries 1–6 at V_D = –60 V. The entry number follows Table 3.

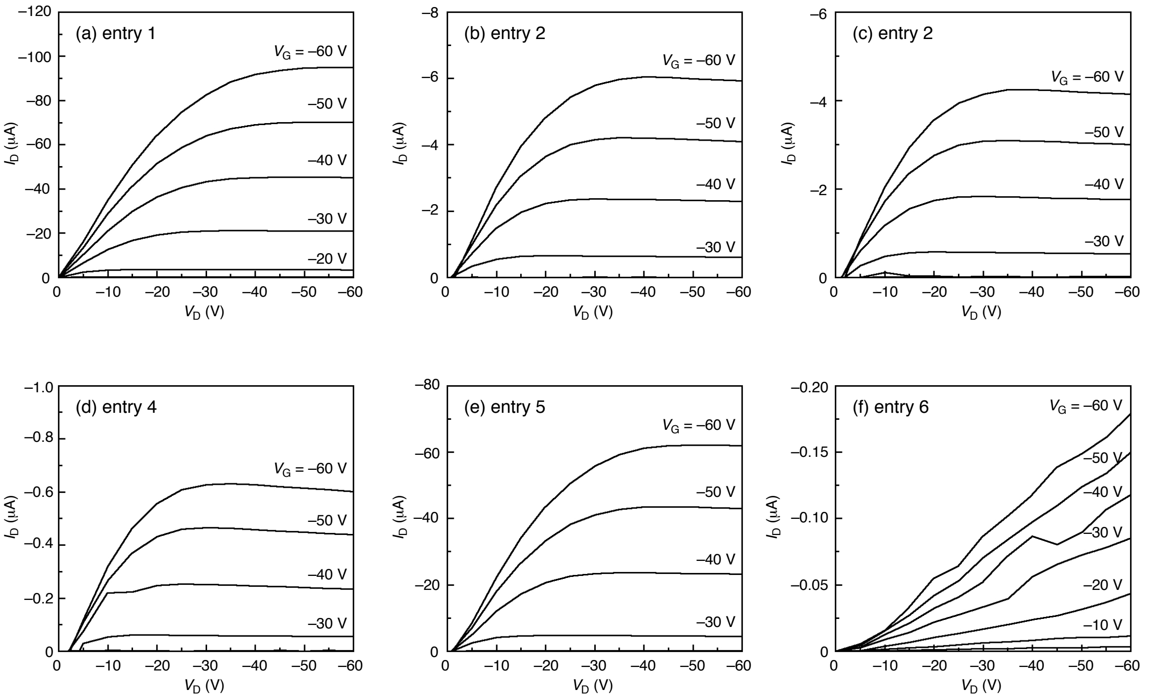

Figure S3. Output characteristics of the OFETs based on poly(**1**-*alt*-**2**) of entries 1–6. The entry number follows Table 3.

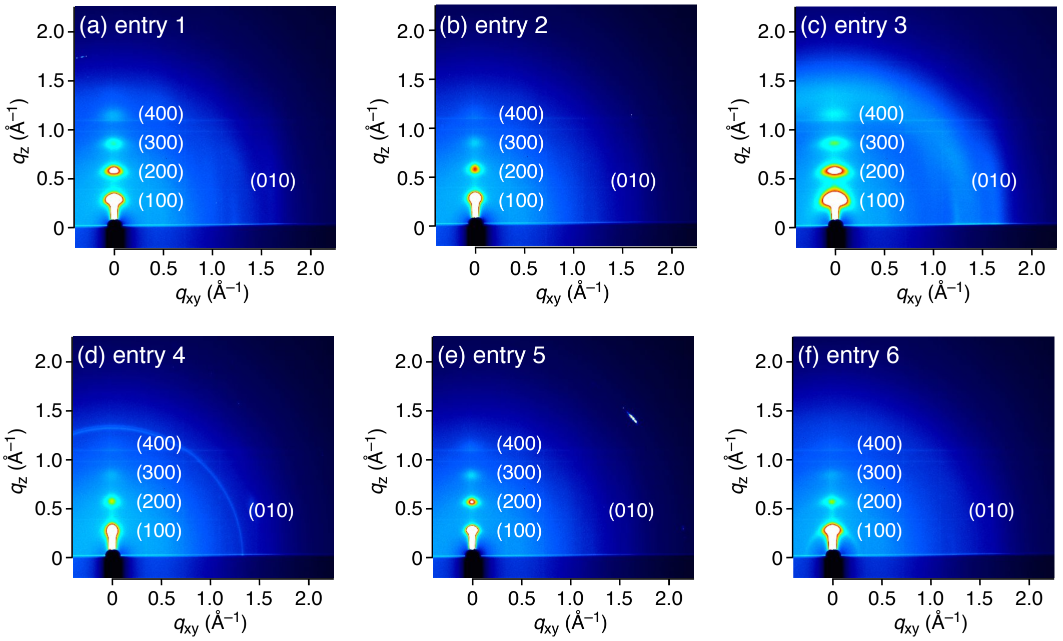

Figure S4. GIWAXS images of the thin films of poly(**1**-*alt*-**2**) of entries 1–6. The entry number follows Table 3.

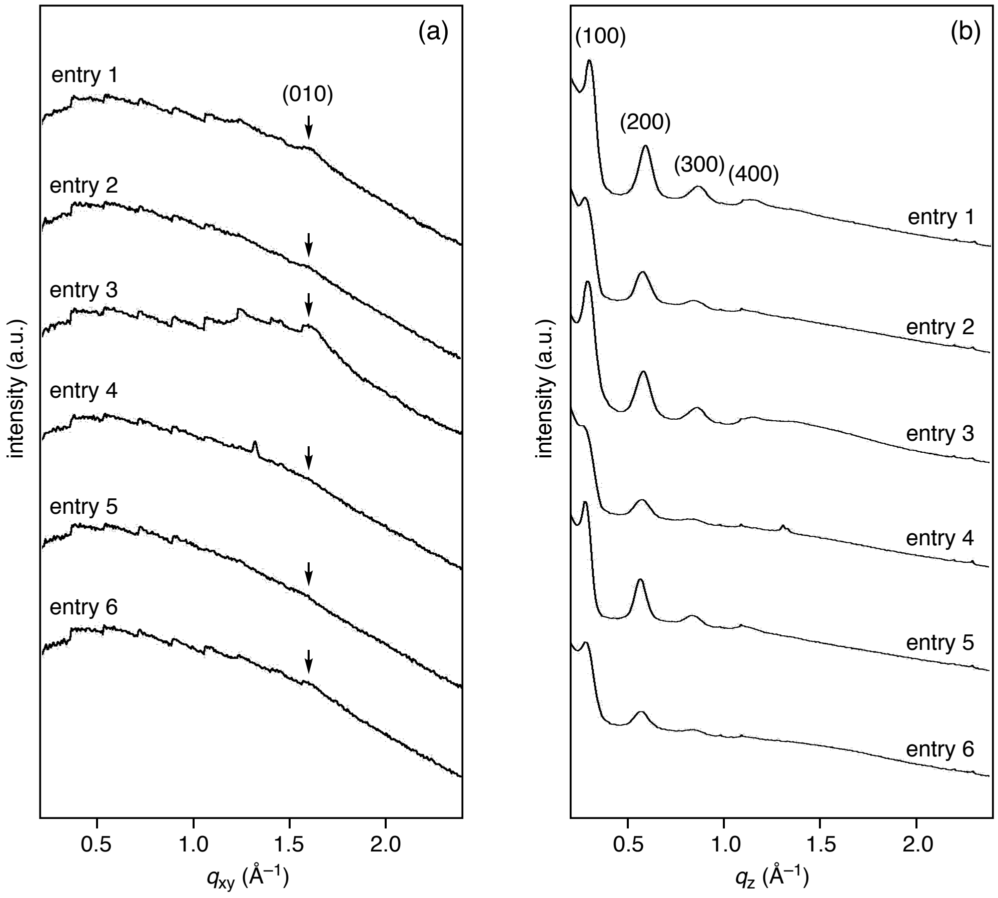

Figure S5. In-plane (a) and out-of-plane (b) line-cuts of GIWAXS measurement for the thin films of poly(**1**-*alt*-**2**) of entries 1–6. The entry number follows Table 3.

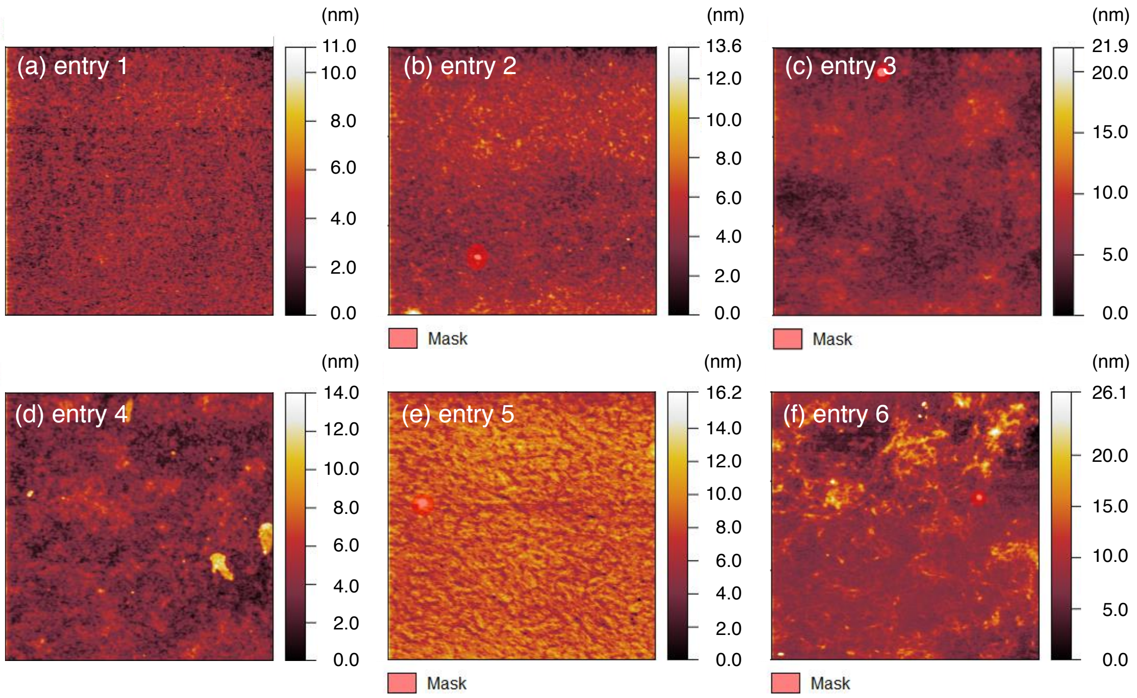

Figure S6. AFM images (6 × 6 μm) of the thin films of poly(**1**-*alt*-**2**) of entries 1–6. The entry number follows Table 3.

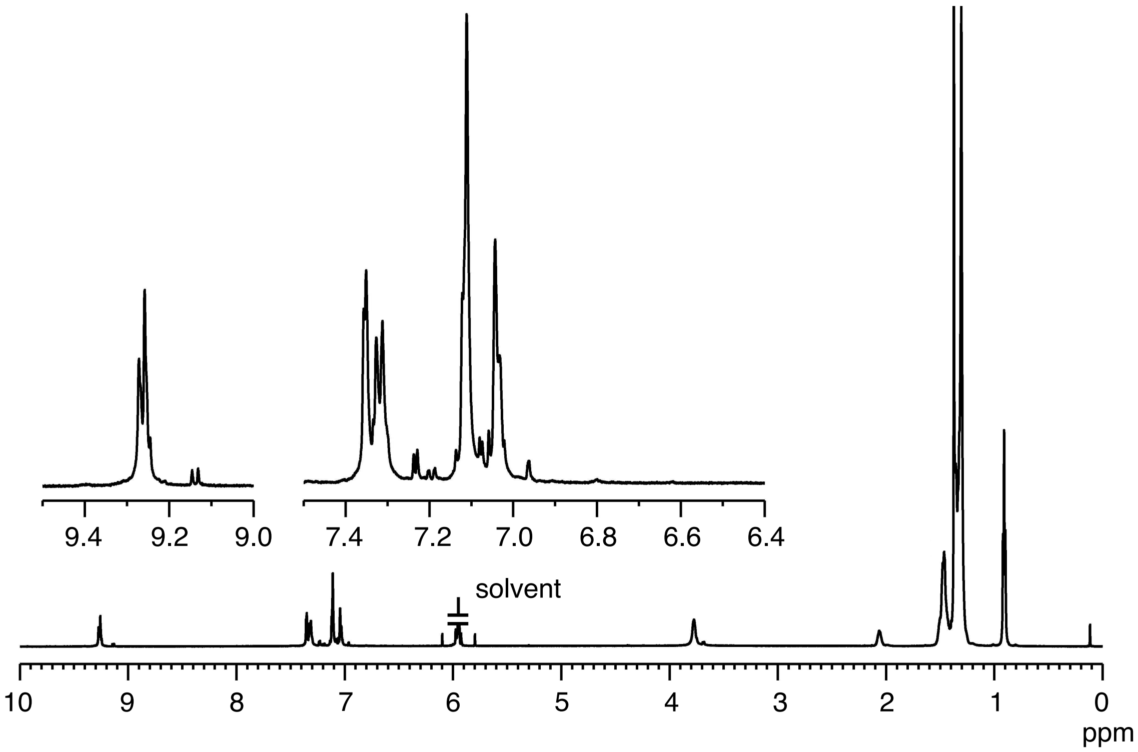

Figure S7. ^{1}H NMR spectrum of poly(**1**-*alt*-**2**) prepared via DArP (M_n = 15,700; entry 5 in Table 1, entry 1 in Table 3) in $C_2D_2Cl_4$ at 130 °C (600 MHz).

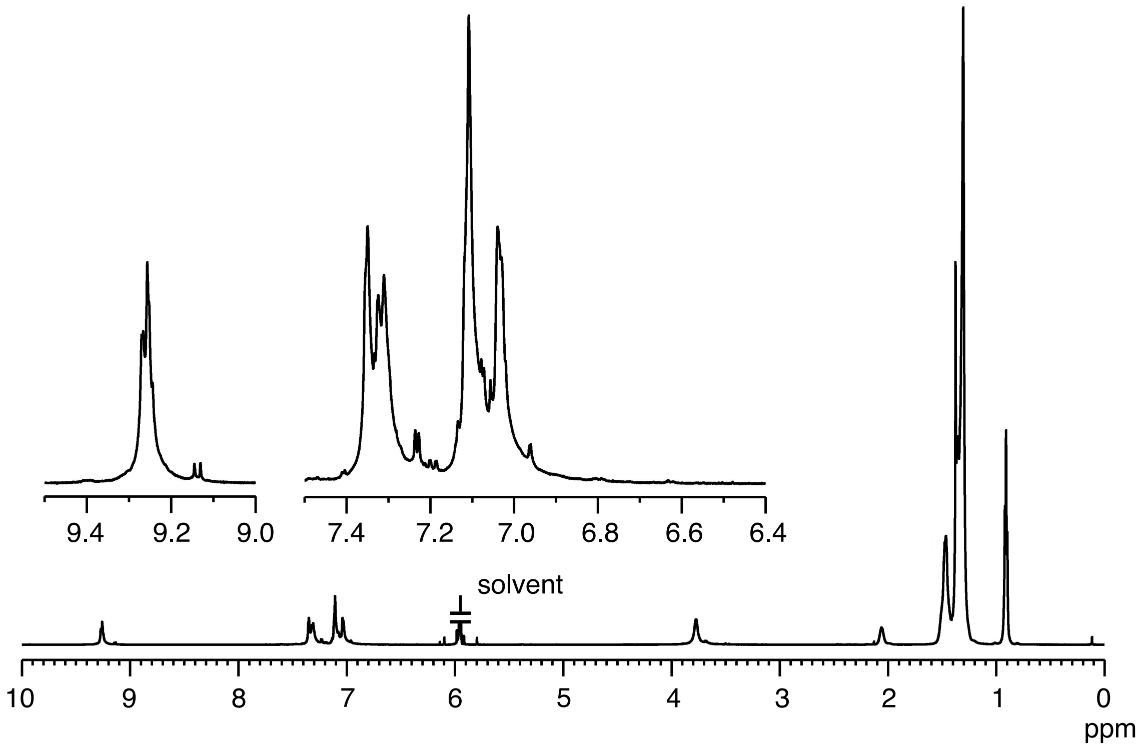

Figure S8. ¹H NMR spectrum of poly(**1**-*alt*-**2**) prepared via DArP (M_n = 17,000; entry 1 in Table 1, entry 2 in Table 3) in $C_2D_2Cl_4$ at 130 °C (600 MHz).

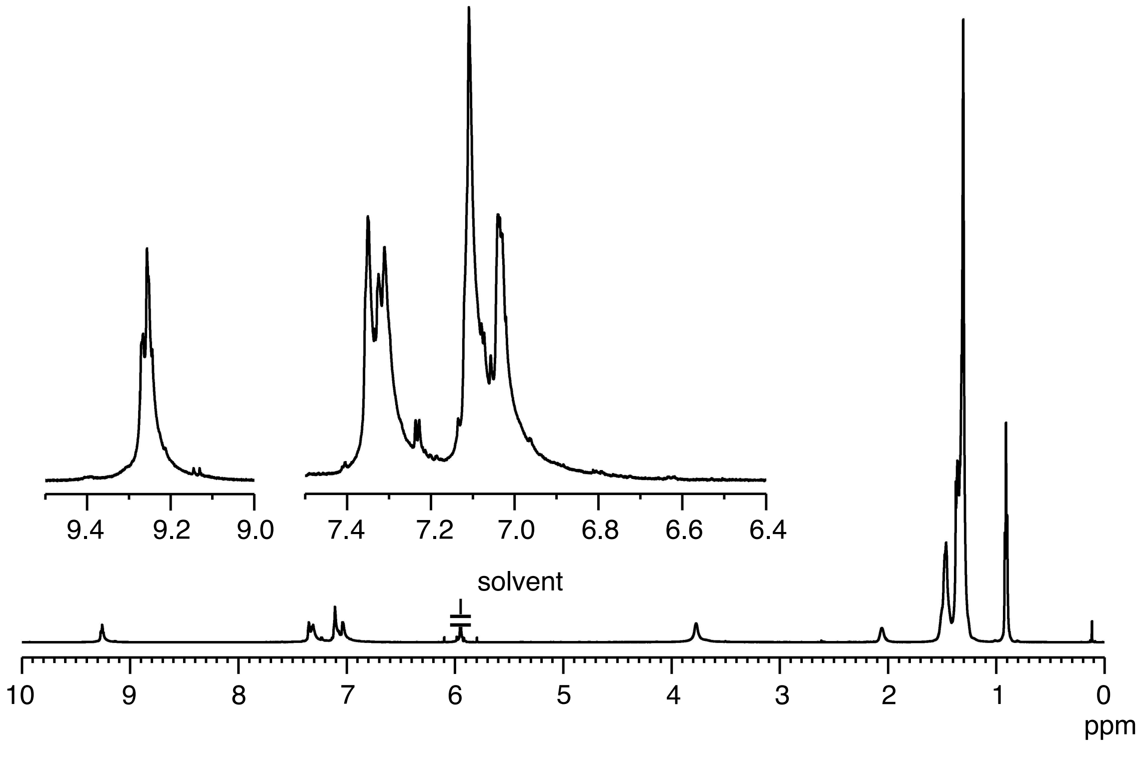

Figure S9. ¹H NMR spectrum of poly(**1**-*alt*-**2**) prepared via DArP (M_n = 22,700; entry 3 in Table 1 and Table 3) in $C_2D_2Cl_4$ at 130 °C (600 MHz).

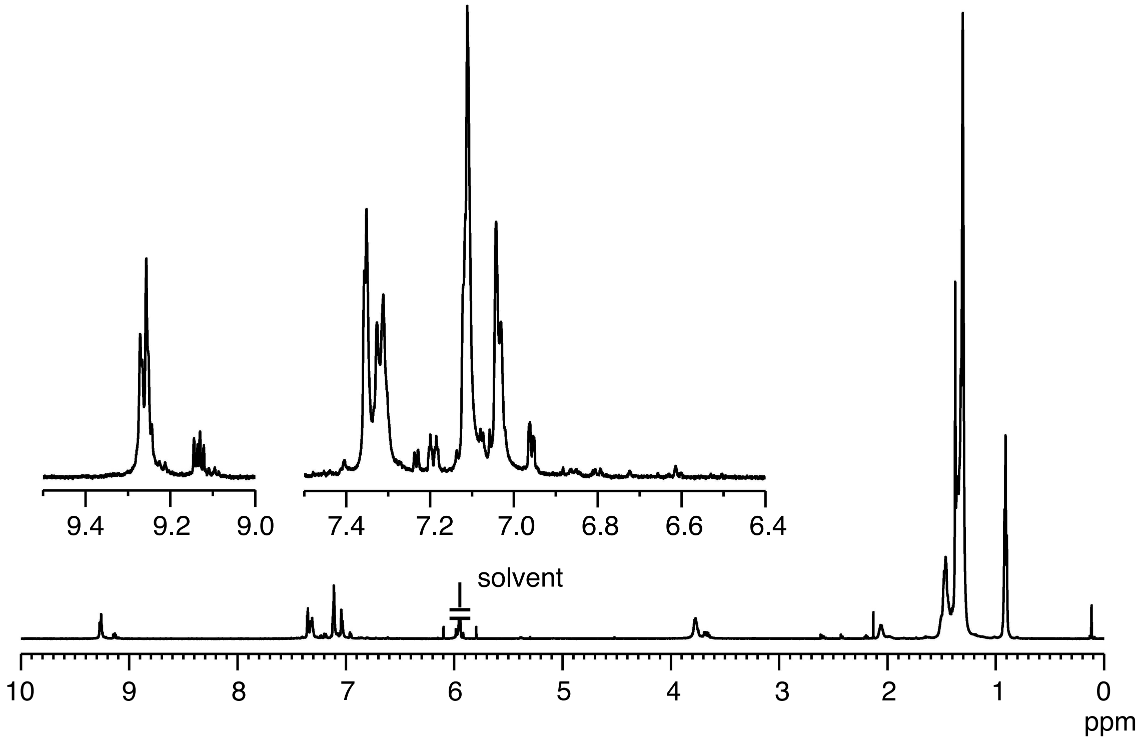

Figure S10. ^{1}H NMR spectrum of poly(**1**-*alt*-**2**) prepared via Migita–Kosugi–Stille cross-coupling polymerization (M_n = 17,700; entry 5 in Table 3) in $C_2D_2Cl_4$ at 130 °C (600 MHz).

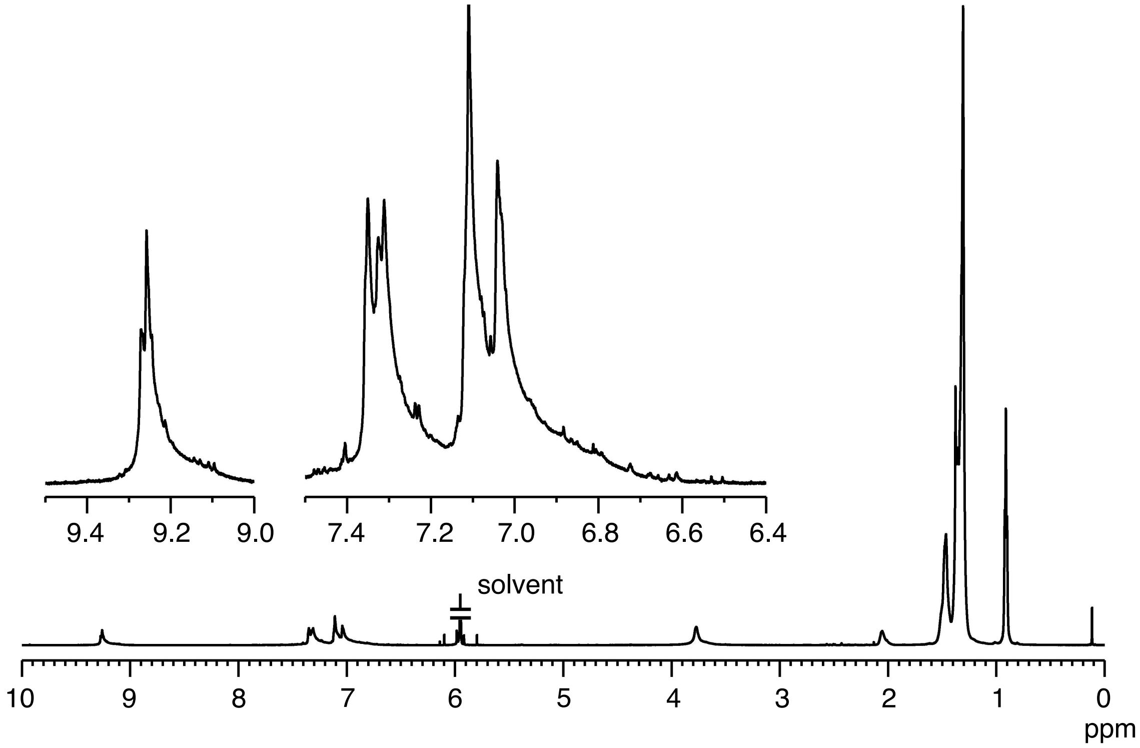

Figure S11. ^{1}H NMR spectrum of poly(**1**-*alt*-**2**) prepared via Migita–Kosugi–Stille cross-coupling polymerization (M_n = 24,400) in $C_2D_2Cl_4$ at 130 °C (600 MHz).

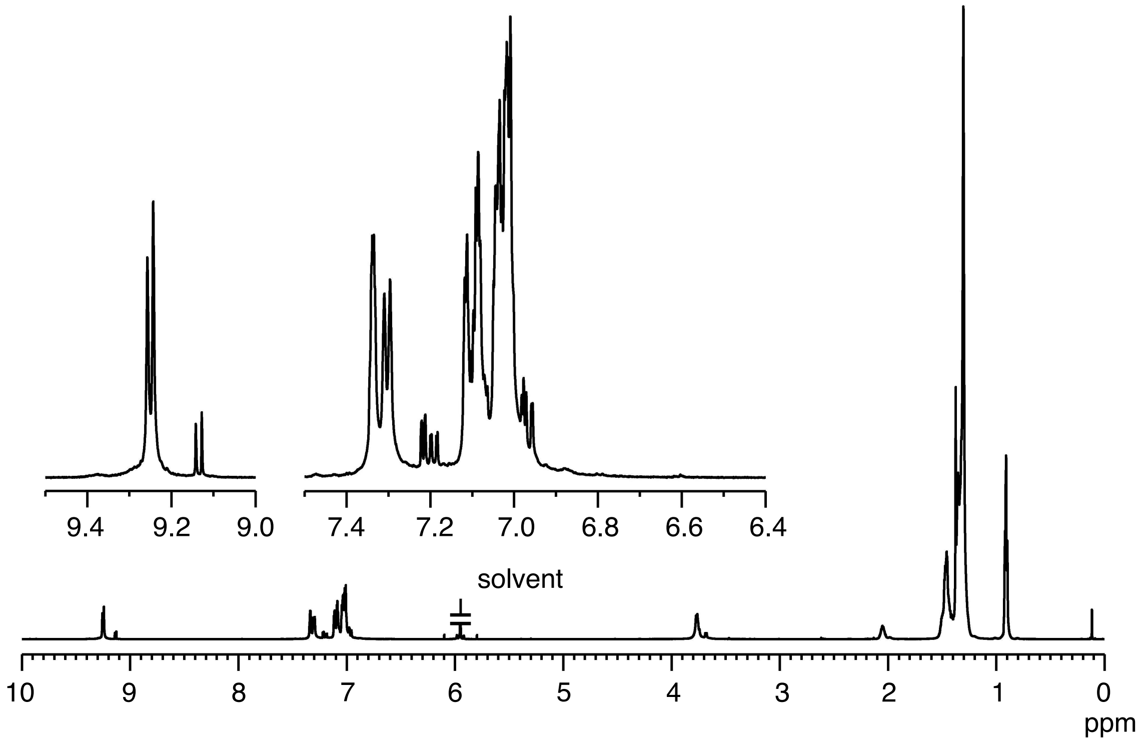

Figure S12. ^{1}H NMR spectrum of poly(**1**-*alt*-**2**$_2$) in $C_2D_2Cl_4$ at 130 °C (600 MHz).
